# Comparative Conformational Sampling of Apo Adenylate Kinase by Enhanced Molecular Dynamics and a Sequence-Based Generative Ensemble Model

**DOI:** 10.3390/molecules31203686

**Published:** 2026-10-14

**Authors:** Qingling Zhao, Ting Cui, Ying Li

**Affiliations:** 1 Library of Beijing Union University, Beijing 100101, China; 2 College of Biochemical Engineering, Beijing Union University, Beijing 100023, China

**Keywords:** conformational sampling, adenylate kinase, metadynamics, targeted MD, Gaussian accelerated MD, simulated tempering, BioEmu, molecular dynamics, open–closed transition

## Abstract

Conformational sampling remains a major challenge in molecular dynamics (MD) simulations of proteins with large-scale functional motions. Here, we use apo *Escherichia coli* adenylate kinase (AdK) as a benchmark for the open–closed transition and compare nine sampling protocols under a uniform explicit-solvent budget of 300 ns per method. The methods include unbiased MD, metadynamics, targeted MD (TMD), accelerated MD, simulated tempering, interpolation-based MD, and BioEmu. Closed-state sampling was evaluated relative to a reference-closed basin defined from ligand-stripped PDB 1AKE. TMD was the only method that both reached and maintained the closed reference, whereas unbiased MD did not enter this basin within 300 ns. Metadynamics increased sampling along the domain-distance coordinate but also promoted excessive LID opening, while simulated tempering showed a similar tendency. BioEmu generated closed-like conformations, but these states were not maintained during subsequent short MD simulations. Overall, the results show that collective-variable coverage does not necessarily correspond to stable target state occupancy and that different sampling methods provide different types of conformational information. These findings highlight the importance of selecting sampling strategies according to the specific scientific objective.

## 1. Introduction

Molecular dynamics (MD) has become an important computational approach for studying protein motions. A crystal or predicted structure represents a single conformational snapshot [1], whereas catalysis, allostery, and molecular recognition depend on thermally accessible conformational ensembles and the pathways connecting them [2,3,4]. Explicit-solvent MD provides a physically motivated Hamiltonian, atomic-level temporal resolution, and a direct framework for calculating observables that can be compared with experimental measurements. Advances in GPU computing and molecular force fields have also reduced the practical cost of atomistic simulations, making several hundred nanoseconds of simulation for a medium-sized globular protein feasible on a single consumer GPU [5,6,7]. However, these advances have not eliminated the fundamental sampling problem. Functionally important conformational rearrangements in proteins can occur on microsecond-to-millisecond timescales [6,7]. Consequently, unbiased trajectories of a few hundred nanoseconds may not capture rare but functionally relevant states, and completion of an MD simulation does not by itself demonstrate adequate sampling of the relevant conformational space.

A range of enhanced sampling approaches has therefore been developed, with different requirements, computational costs, and interpretations. Well-tempered metadynamics introduces a history-dependent bias to explore a predefined collective-variable (CV) space and can, in principle, be reweighted to obtain a free-energy surface (FES) [8,9,10,11]. Its performance, however, depends strongly on the choice of CVs, and biased populations should not be interpreted as free-energy differences without appropriate convergence and reweighting [12]. Gaussian accelerated MD (GaMD) enhances sampling by modifying the potential energy landscape without requiring an explicit CV [13,14]. This makes it attractive when an appropriate reaction coordinate is unknown, although the applied boost may be insufficient to overcome high barriers and quantitative interpretation still requires reweighting. Simulated tempering and replica-exchange methods promote barrier crossing through temperature variation [15,16]. These approaches do not require predefined CVs, but elevated temperatures may also enhance motions that are not directly related to the functional transition of interest, and the resulting ensemble requires appropriate reweighting before being interpreted as a 300 K equilibrium ensemble. Targeted MD (TMD) and related path-based methods drive a system toward a predefined target structure and can provide information about feasible transition pathways [17,18]. Such methods require prior structural knowledge of the target state and describe nonequilibrium pathways rather than equilibrium populations. More recently, sequence-based generative ensemble models such as BioEmu have provided a rapid means of generating alternative protein conformations directly from amino-acid sequencing [1,19,20,21]. Although these models offer an efficient way to generate candidate conformations, their thermodynamic interpretation and the stability of generated conformations during subsequent explicit-solvent MD remain important questions.

Despite the diversity of available approaches, these methods are rarely compared systematically on the same protein using the same force field, solvent environment, simulation budget, and analysis criteria. Comparisons based on published studies are often complicated by differences in system size, ligand state, simulation length, and evaluation metrics. A controlled comparison can therefore help clarify what different sampling strategies can achieve under comparable computational conditions and, importantly, what conclusions can reasonably be drawn from their outputs.

*Escherichia coli* adenylate kinase (AdK) provides a suitable benchmark for such a comparison. This 214-residue enzyme undergoes a large open–closed transition involving the LID and NMP domains relative to the CORE domain [22,23]. The open apo structure (PDB 4AKE) and the inhibitor-bound structure 1AKE, used here after removal of the ligand, differ by approximately 7 Å in global Cα RMSD [22,23]. We use proximity to ligand-stripped 1AKE to define a reference-closed basin (hereafter, the closed reference). Although 1AKE is derived from a ligand-bound structure and therefore does not represent an experimentally determined apo closed state, it provides a well-defined structural reference for evaluating conformational sampling. Previous NMR, single-molecule FRET, and computational studies have shown that the LID and NMP domains can undergo partially independent motions and that apo AdK can sample elements of the closing transition, although these events are relatively rare on nanosecond timescales [24,25,26,27,28,29,30,31,32]. Thus, AdK is sufficiently challenging to expose differences among sampling strategies while remaining computationally tractable for systematic comparison.

In this work, we compare nine sampling protocols for apo AdK, with each method receiving 300 ns of production MD following a common minimization and equilibration procedure. The methods span a range of prior structural information, from unbiased MD, GaMD, and simulated tempering, through generic or deliberately nonfunctional CVs, functional domain-distance CVs, a predefined closed reference, Cartesian interpolation, and BioEmu as a sequence-based generative ensemble model. All trajectories are evaluated using a common set of measures, including occupancy of the NMP–CORE/LID–CORE conformational plane, RMSD relative to the open and closed reference structures, the order of LID and NMP closure, coverage growth, Cα flexibility, and computational cost. Because the biased simulations were not reweighted to a converged FES, we do not interpret the resulting biased distributions as an open–closed free-energy difference. Instead, the aim is to characterize the distinct sampling behavior of these approaches and to clarify which types of conformational information each method can provide under a common computational budget.

## 2. Results

### 2.1. Closed-State Occupancy and Domain-Plane Coverage Provide Distinct Assessments of Sampling

The nine protocols produced markedly different conformational ensembles under the same nominal 300 ns production budget (Table 1). TMD achieved the closest approach to the closed reference, with a minimum Cα RMSD of 1.28 Å and 54.88% closed-like frames. Blind metadynamics and well-tempered metadynamics reached minimum RMSDs of 2.43 and 2.89 Å, respectively, but produced closed-like frames in only 0.93% and 0.43% of the trajectory. Interpolation MD and BioEmu followed by MD relaxation produced minimum RMSDs of 3.06 and 3.31 Å, respectively, whereas unbiased MD, wrong-CV metadynamics, and dihedral GaMD remained close to the open ensemble. Simulated tempering showed the largest minimum RMSD from the closed reference (5.83 Å).

These results also show that domain-plane coverage and closed-state occupancy provide different assessments of sampling. Functional metadynamics occupied the largest number of CV bins (1260), whereas TMD occupied only 213 bins, similar to unbiased MD (212 bins). The narrow coverage of TMD reflects its directed trajectory rather than poor sampling of the target state. Thus, the number of occupied CV bins alone does not indicate how effectively a protocol samples a predefined functional conformation.

### 2.2. Metadynamics Expands the Domain-Distance Map Mainly Through LID Over-Opening

**Figure 1:**
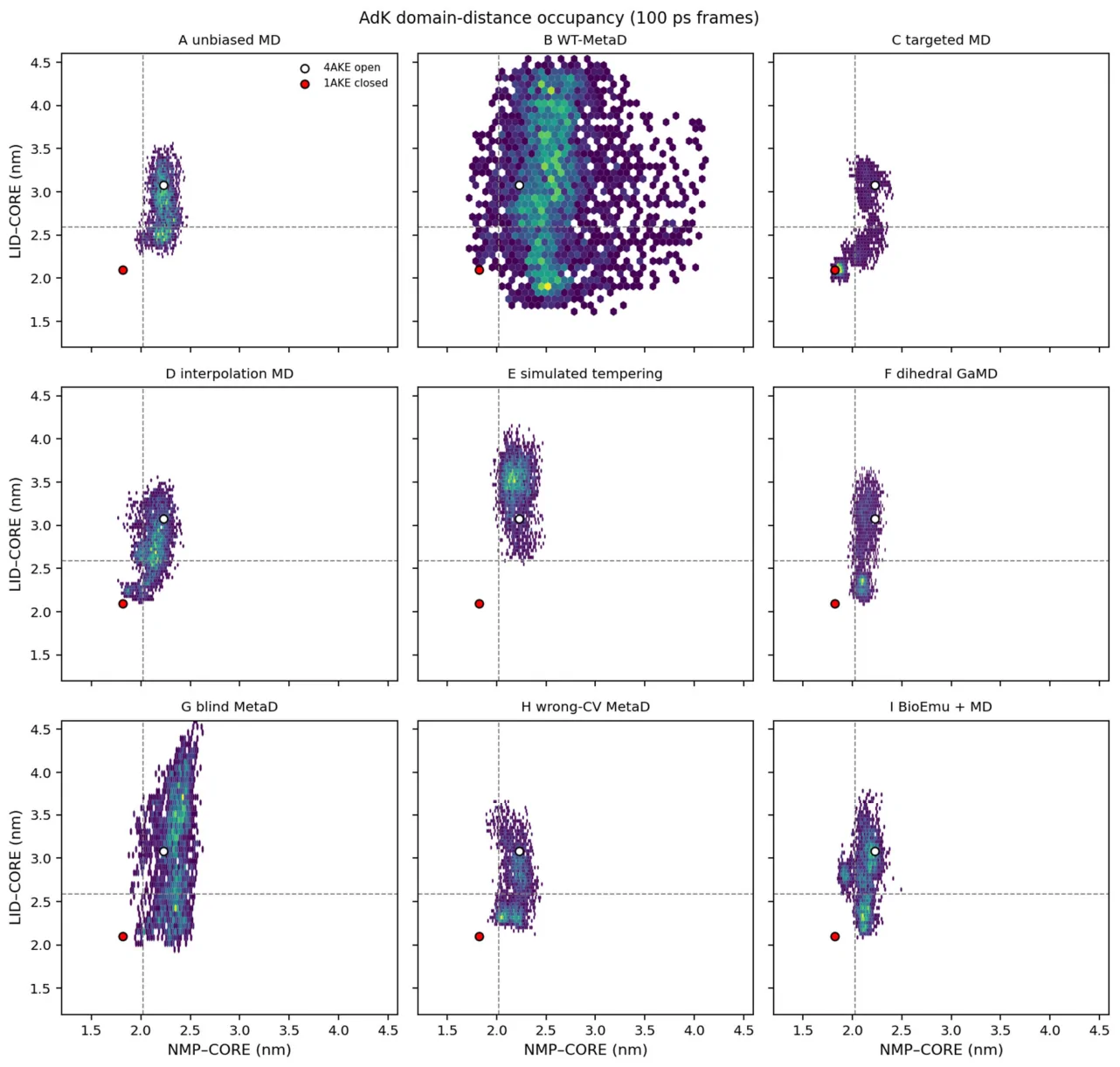
Occupancy of the NMP–CORE/LID–CORE plane for representative methods. Distances are Cα centers of mass, sampled every 100 ps. White and red markers indicate the 4AKE and ligand-stripped 1AKE crystals; Hexagon color indicates the number of sampled frames in each bin, from low occupancy (dark purple) to high occupancy (yellow); dashed lines mark the midpoints of the crystal distances. Functional metadynamics fills the plane, including over-open LID geometries, whereas TMD traces a narrow open-to-closed corridor. Methods D and I concatenate six 50 ns seeds.

The biologically relevant conformational change in AdK involves movements of the NMP and LID domains toward the CORE domain. Figure 1 shows the occupancy of this two-dimensional domain-distance space. Unbiased MD remained concentrated near the open crystal, with mean LID–CORE and NMP–CORE distances of approximately 2.84 and 2.23 nm, respectively, and occupied 212 bins after 300 ns. TMD followed a relatively narrow trajectory from the open state toward the closed reference (1.82 nm for NMP–CORE and 2.10 nm for LID–CORE) and subsequently remained near the closed state. Its total coverage was therefore similar to that of unbiased MD.

In contrast, functional metadynamics occupied 1260 bins, with LID–CORE distances reaching 4.54 nm and NMP–CORE distances reaching 4.11 nm, extending well beyond the regions defined by the two crystal structures. Blind metadynamics provided the broadest coverage among methods that did not use the domain-distance map, occupying 494 bins, but also produced substantial LID opening, with a maximum LID–CORE distance of 4.67 nm. Simulated tempering similarly increased LID–CORE distances, with a mean of 3.45 nm and a maximum of 4.15 nm, while producing comparatively little movement of the NMP domain. Dihedral GaMD occupied 198 bins, slightly fewer than unbiased MD.

**Figure 2:**
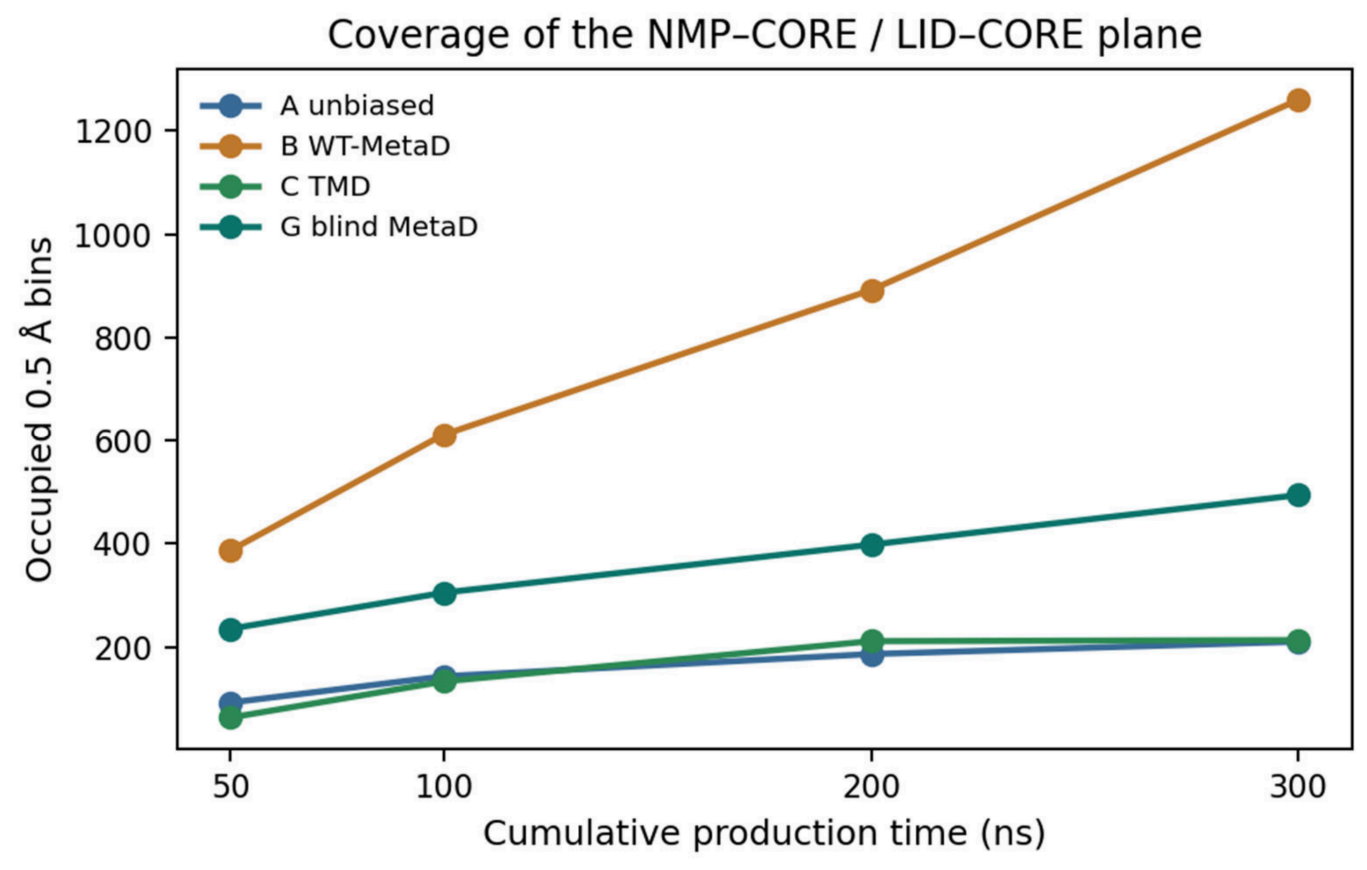
Growth of occupied 0.05 nm bins on the NMP–CORE × LID–CORE plane for four representative methods. Functional metadynamics occupies 387 bins by 50 ns, already more than the 50 ns coverage of the other methods. TMD remains a thin corridor. Time series and coverage for all nine methods are shown in Supplementary Figures S1–S4.

Coverage as a function of accumulated simulation time further emphasized the difference between exploration and target state sampling (Figure 2). Functional metadynamics occupied 387 bins within the first 50 ns, compared with 235 bins for the next most extensive method at the same time point, and already exceeded the full 300 ns coverage of every protocol except blind metadynamics. Blind metadynamics remained the second-highest coverage protocol throughout the simulation. TMD accumulated bins more slowly because its trajectory remained concentrated along a narrow open-to-closed pathway.

### 2.3. Only TMD Reaches and Maintains the Closed Reference

**Figure 3:**
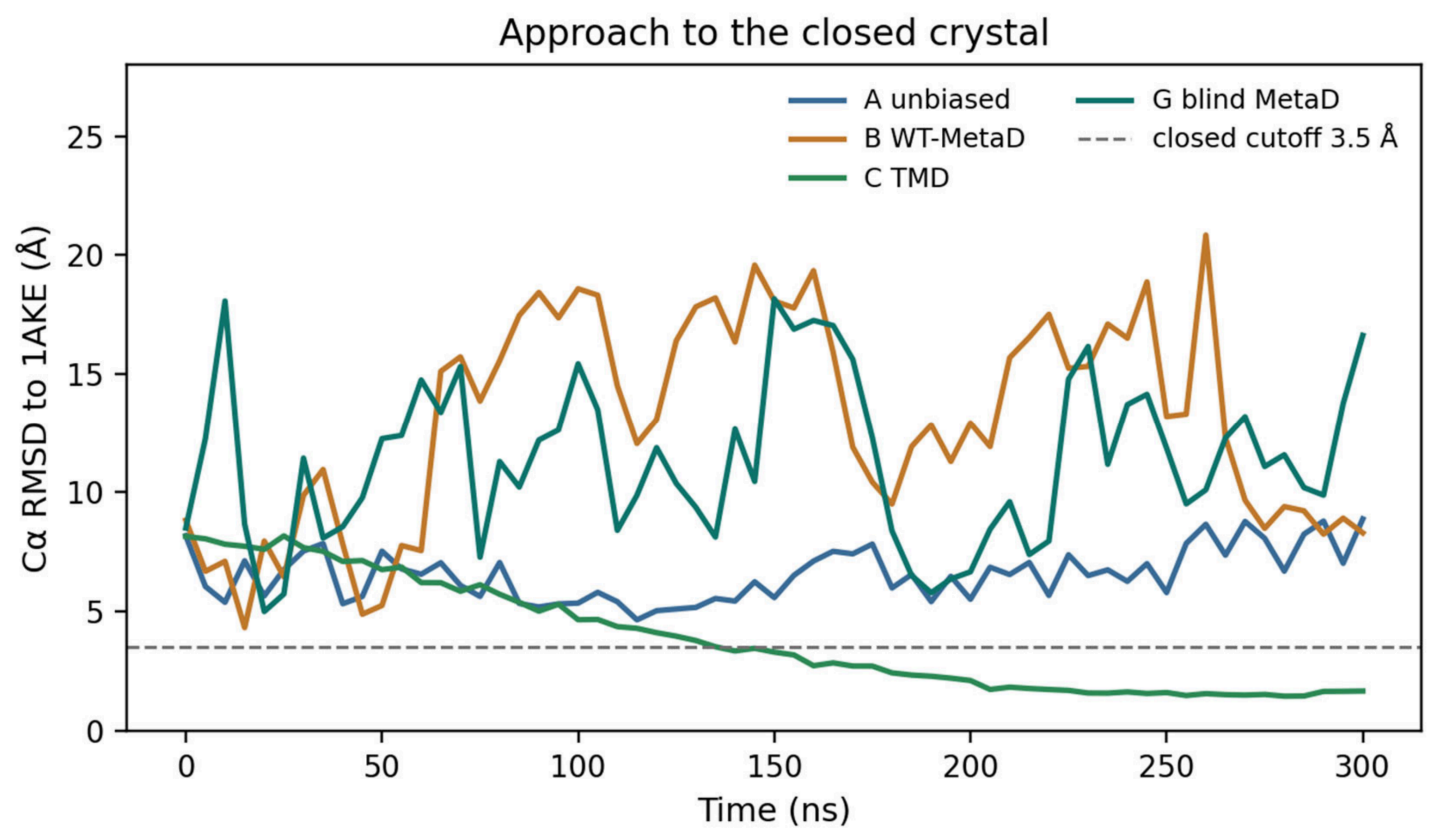
Global Cα RMSD to ligand-stripped 1AKE for representative methods (frames saved every 100 ps and plotted every 5 ns). Only TMD remains below the 3.5 Å closed-reference cutoff; unbiased MD never enters that window, and metadynamics visits it only transiently. RMSD to 4AKE and the remaining methods are shown in Supplementary Figures S1–S3.

Proximity to the closed reference provides a more stringent measure of target state sampling than CV-plane coverage because highly open conformations can contribute substantially to CV coverage while remaining distant from both reference structures. Figure 3 shows the global Cα RMSD to ligand-stripped 1AKE for representative methods.

Unbiased MD fluctuated between approximately 4 and 9 Å and never crossed the 3.5 Å closed-state threshold. Its minimum RMSD was 3.86 Å, and 81.5% of frames remained open-like relative to 4AKE. In contrast, TMD approached the closed reference almost monotonically, crossed the 3.5 Å threshold at 128.7 ns, and remained near 1.5 Å after approximately 200 ns. The minimum RMSD was 1.28 Å, with 54.9% of frames classified as closed-like and a final RMSD of 1.57 Å.

**Figure 4:**
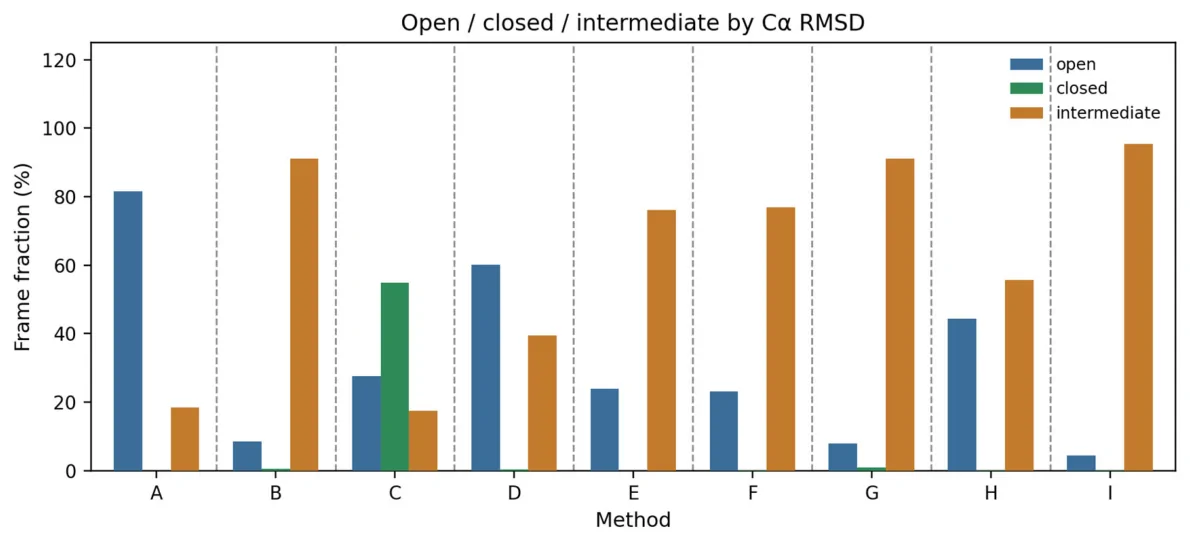
Frame fractions classified as open-like (Cα RMSD < 3.5 Å to 4AKE), closed-like (Cα RMSD < 3.5 Å to ligand-stripped 1AKE), or intermediate. Closed-like frames are the majority only for TMD; metadynamics is dominated by intermediate, over-open states. Vertical dashed lines separate methods A–I.

Frame fractions classified as open-like, closed-like, or intermediate are summarized in Figure 4; closed-like frames are the majority only for TMD. Both functional and blind metadynamics produced transient approaches to the closed reference, with first crossings at 20.8 and 20.7 ns, respectively. However, closed-like frames accounted for only 0.43% and 0.93% of their trajectories. At later times, their RMSDs increased to 8–20 Å, consistent with progressive LID/NMP over-extension rather than sustained progression toward the closed reference. Simulated tempering, GaMD, interpolation MD, wrong-CV metadynamics, and BioEmu followed by MD relaxation likewise failed to maintain stable closed-state occupancy (Figures S1 and S2).

**Figure 5:**
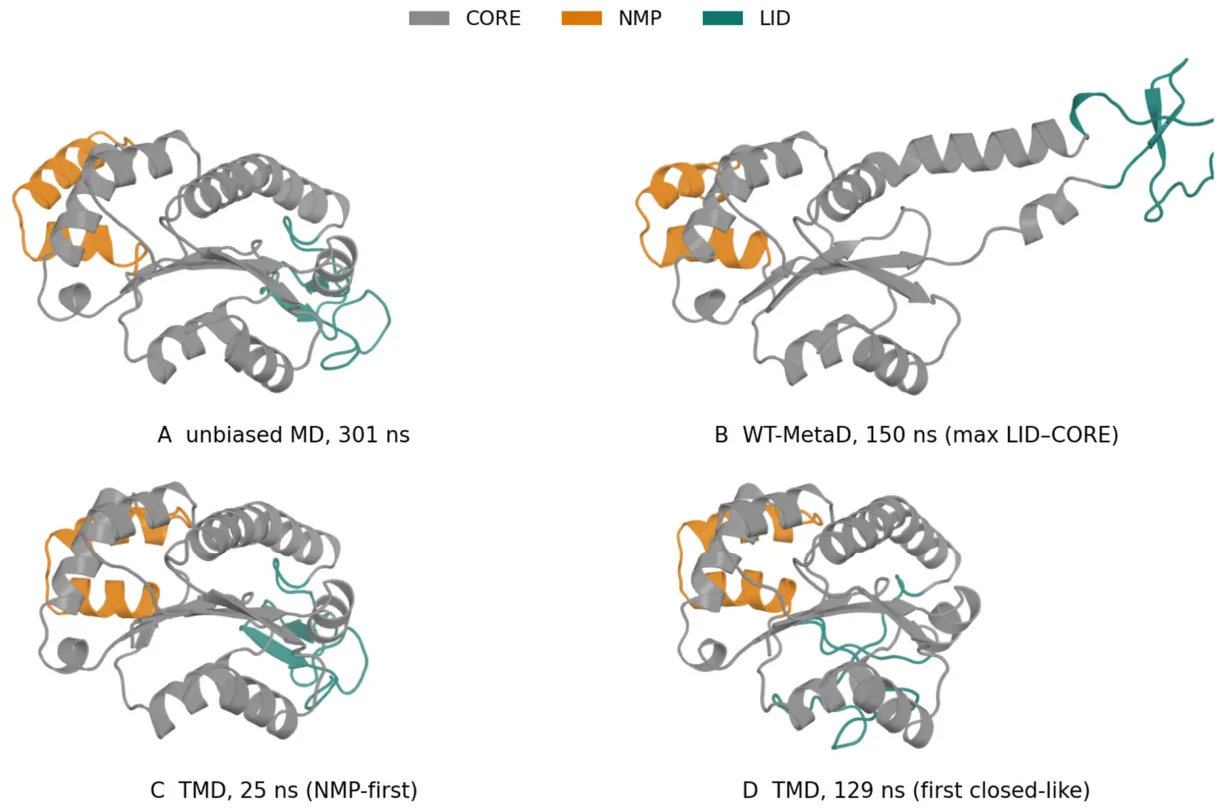
Domain-colored snapshots from the 300 ns open-start trajectories of methods A–C. (**A**) Unbiased MD at 301 ns remains open-like. (**B**) Well-tempered metadynamics at 150 ns, the frame of maximum LID–CORE distance, showing LID over-opening. (**C**) Targeted MD at 25 ns, after the NMP domain has crossed its midpoint. (**D**) Targeted MD at 129 ns, the first closed-like frame (Cα RMSD to ligand-stripped 1AKE < 3.5 Å). Cartoons are CORE-aligned to 4AKE in a common camera. CORE, gray; NMP, orange; LID, teal. Closed-start 1 µs and reverse-TMD cartoons are shown in Supplementary Figures S13–S16. Methods D–I are shown in Figure S17.

The structural snapshots in Figure 5 provide a corresponding view of these trajectories. Unbiased MD remained close to the open state, whereas metadynamics produced pronounced LID opening. TMD first brought the NMP domain across its midpoint and subsequently reached a closed-like conformation while the RMSD restraint remained active. Corresponding cartoons for methods D–I and for the closed-start 1 µs and reverse-TMD runs are shown in Supplementary Figures S13–S17.

**Figure 6:**
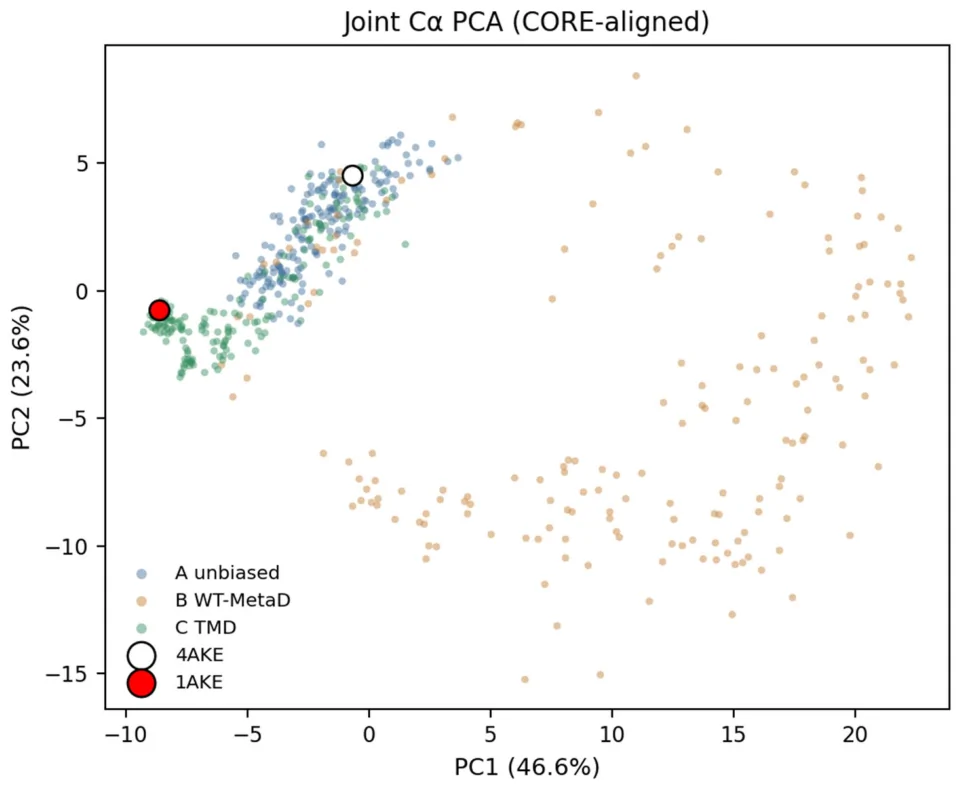
Joint Cα PCA after CORE superposition onto 4AKE (250-frame samples). TMD reaches the closed-reference region, whereas functional metadynamics spreads along directions that include LID over-opening. White and red markers indicate 4AKE and ligand-stripped 1AKE. Results for all nine methods are shown in Supplementary Figure S6.

Joint PCA after CORE superposition (Figure 6) shows the same distinction in a reduced coordinate space: TMD reaches the region of the closed reference, whereas metadynamics explores a broader region that includes substantial over-opening along the LID coordinate. Nine-method RMSF and PCA overlays are Supplementary Figures S5 and S6.

**Figure 7:**
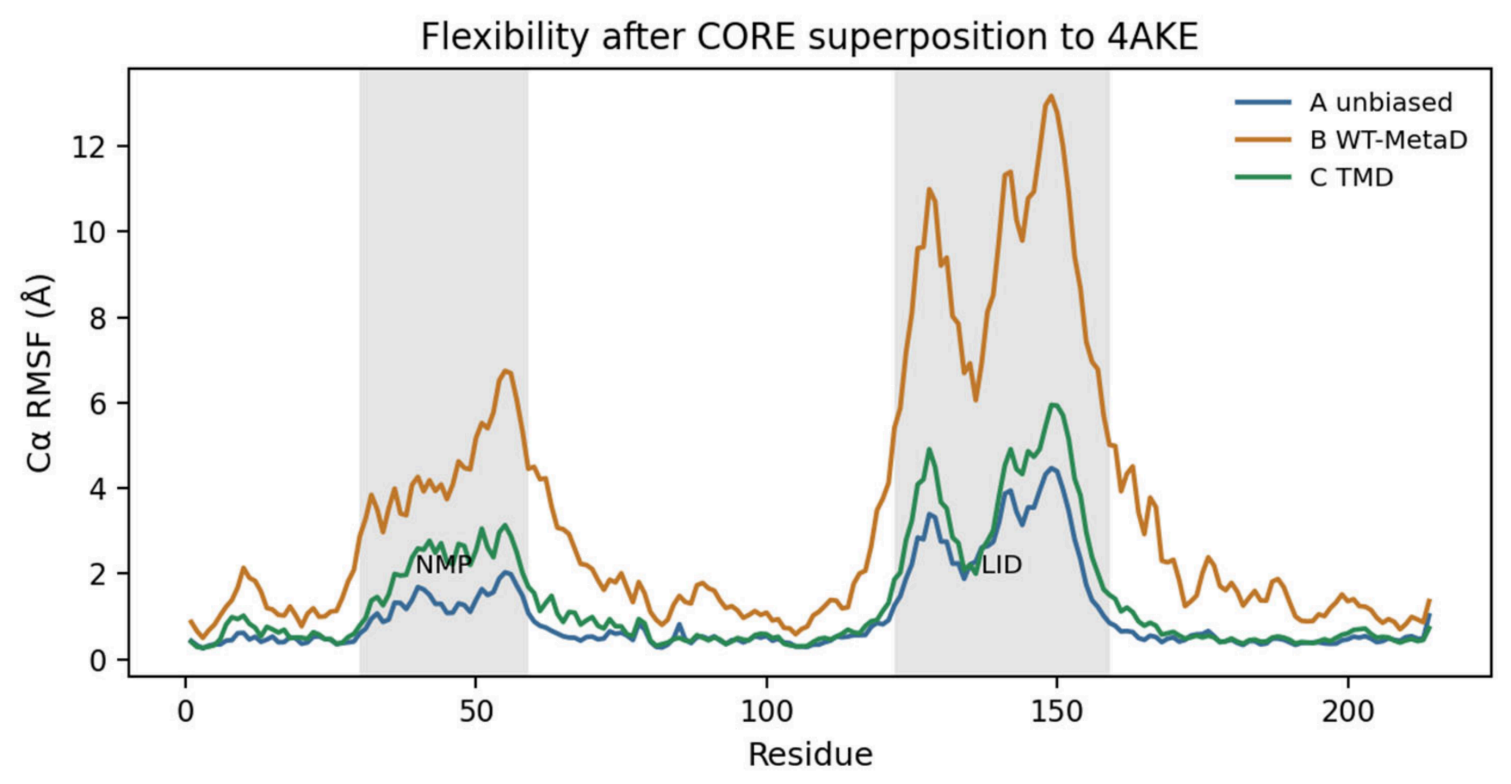
Cα RMSF after CORE superposition onto 4AKE. Shaded intervals mark NMP (residues 30–59) and LID (residues 122–159). Metadynamics inflates LID RMSF well beyond unbiased thermal motion; TMD LID RMSF reflects the driven closing stroke. Remaining methods are shown in Supplementary Figure S5.

Cα RMSF after CORE superposition is shown in Figure 7. Unbiased MD, functional metadynamics, and TMD all have LID > NMP > CORE. Unbiased LID RMSF is 2.74 Å. Functional metadynamics inflates LID RMSF to 9.03 Å, a value that includes nonequilibrium over-opening and should not be quoted as equilibrium flexibility; blind metadynamics is similar (9.45 Å; Figure S5). TMD LID RMSF (3.64 Å) reflects the driven closing stroke plus the hold phase, not a thermal fluctuation of the open enzyme.

### 2.4. Closing Order of the LID and NMP Domains

The relative timing of LID and NMP closure has been extensively studied in AdK [25,26]. Using the midpoint-defined quadrants of the NMP–CORE/LID–CORE plane, unbiased MD showed greater LID mobility than NMP mobility. The CO quadrant, corresponding to a relatively closed LID with an open NMP domain, contained 24.7% of frames, whereas the OC quadrant contained only 0.1%. The CC quadrant accounted for 1.4% of frames, and these frames did not coincide with a Cα RMSD below 3.5 Å to 1AKE. The correlation between NMP–CORE and LID–CORE distances was only 0.09.

TMD followed a different sequence under the global Cα RMSD restraint. The NMP domain crossed its midpoint at 24.6 ns, whereas the LID crossed its midpoint at 83.6 ns. The trajectory reached the 3.5 Å closed-reference threshold at 128.7 ns, after which the CC quadrant accounted for 51.9% of frames. The NMP–LID distance correlation increased to 0.76. Because the TMD restraint acts on all Cα atoms relative to 1AKE, the observed NMP-first sequence represents a kinematically accessible path under the applied restraint rather than direct evidence for the equilibrium mechanism of AdK closure.

Interpolation and BioEmu seeds also produced more mixed-quadrant configurations than unbiased MD, with OC populations of 10.3% and 11.5%, respectively. This behavior is consistent with their starting structures already containing partially closed NMP configurations. Simulated tempering, in contrast, remained predominantly in the OO quadrant (98.7% of frames), indicating that temperature elevation promoted further LID opening rather than stable recruitment of the NMP domain.

### 2.5. Functional and Blind Collective Variables Produce Different Sampling Behavior

Methods B and G were used to examine the influence of prior structural information on metadynamics sampling. Functional domain-distance CVs produced the largest overall coverage, occupying 1260 bins. Blind metadynamics, using only the Cα radius of gyration and RMSD to the starting structure, nevertheless approached the closed reference more closely than functional metadynamics, with minimum RMSDs of 2.43 and 2.89 Å, respectively. The corresponding closed-like frame fractions were 0.93% and 0.43%. Both protocols, however, produced substantial LID over-opening.

The wrong-CV control, which biased two intra-CORE distances unrelated to LID/NMP opening, produced behavior close to the unbiased baseline and is described in Supplementary Section S5 and Figure S9. These comparisons indicate that broad exploration does not necessarily require a functionally meaningful CV, but the resulting distribution depends strongly on the directions in which the applied bias drives the system.

**Figure 8:**
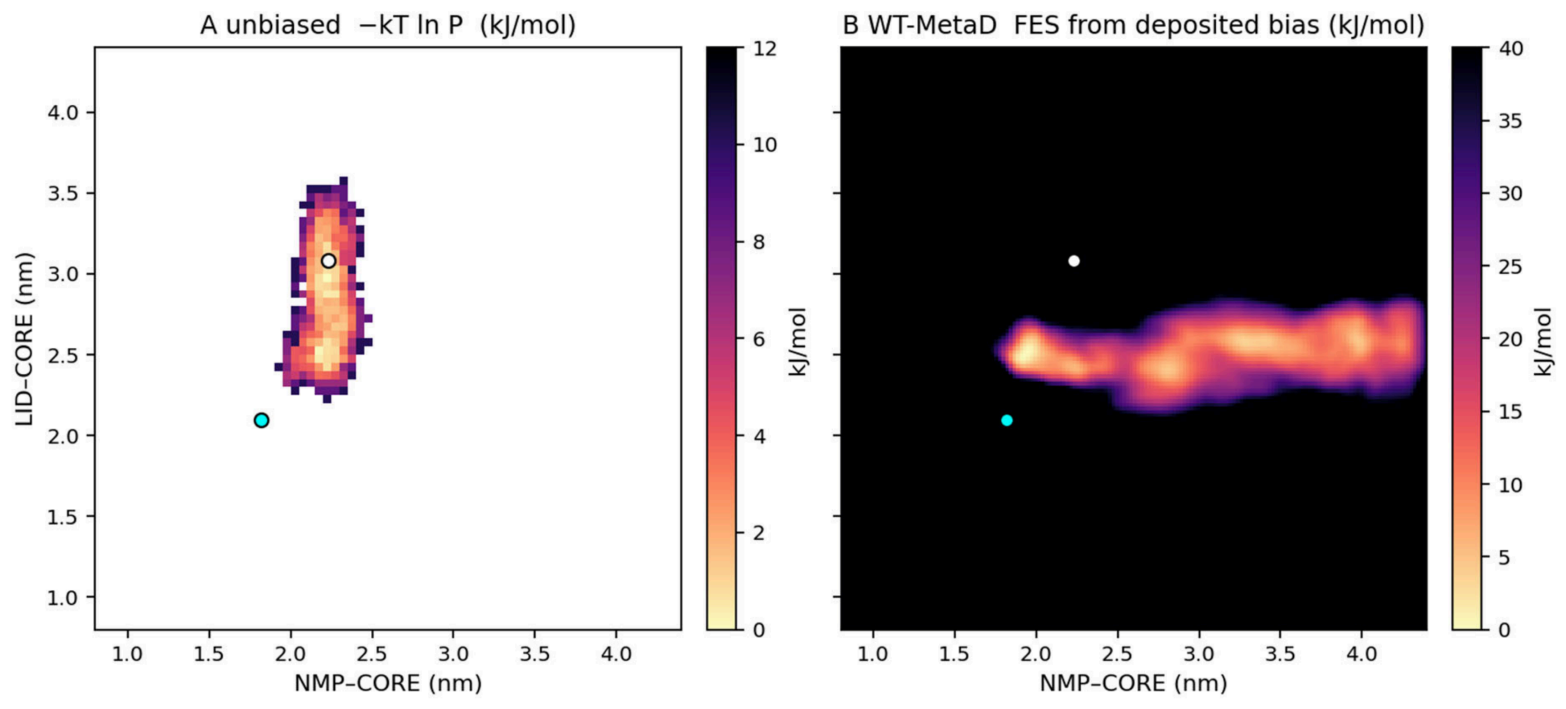
Comparison of unbiased and metadynamics maps on the domain-distance plane. (**A**): Histogram free-energy surface, −*kT* ln *P*, from unbiased occupancy (empty bins were not visited). (**B**): Well-tempered metadynamics FES reconstructed from the deposited bias for method B; the closed reference falls in a poorly sampled region. The metadynamics map is the free energy implied by the bias potential, not a reweighted Δ*G*. White and cyan circles mark the 4AKE (open) and ligand-stripped 1AKE (closed) crystal distances, respectively.

The metadynamics bias was not interpreted as a quantitative free-energy estimate. Reconstruction of an FES from the deposited bias for method B placed the closed reference approximately 46 kJ mol^−1^ above the open crystal. However, the closed-state coordinates were located near a poorly sampled region reaching approximately 129 kJ mol^−1^ over a substantial portion of the grid (Figure 8). Because the biased distribution was not sufficiently converged and was not reweighted, these values should not be interpreted as an open–closed free-energy difference.

### 2.6. Simulated Tempering and Dihedral GaMD Do Not Populate the Closed Reference

Simulated tempering produced the lowest degree of closed-state sampling among the tested protocols. All eight temperature levels were visited, with approximately 18% of samples at 300 K, 9% at 400 K, and 7–16% at the intermediate temperatures (Supplementary Figure S8). The mean production temperature was 344 K, and the potential energy was approximately 30,000 kJ mol^−1^ higher than in the 300 K simulations, indicating that the trajectory represented a temperature mixture rather than a 300 K ensemble.

The elevated temperature increased LID RMSF to 4.70 Å and increased the LID–CORE distance while reducing proximity to 1AKE. The 300–400 K temperature range was sufficient to promote substantial LID opening but did not produce stable CC occupancy within the 300 ns budget. Because the trajectory was not reweighted to 300 K, its conformational distribution cannot be directly interpreted as a 300 K equilibrium ensemble.

Dihedral GaMD similarly did not increase domain-plane coverage relative to unbiased MD, occupying 198 versus 212 bins. The trajectory approached the closed-reference threshold only once, at 199.7 ns, with a minimum RMSD of 3.48 Å and a closed-like fraction of 0.03%. GaMD did increase occupancy of the CO quadrant to 40.1%, indicating that the boost affected the conformational distribution, but it did not produce stable closed-state occupancy under the present simulation conditions. The applied boost was relatively mild (*k*_0_ = 0.107), and stronger boosting would require a separate assessment of sampling and reweighting.

### 2.7. Closed-like BioEmu and Interpolation Seeds Do Not Persist During Short Unbiased MD

BioEmu was the only sequence-based generative ensemble model included in the comparison. The six extracted models were predominantly closed-like relative to 1AKE, with Cα RMSDs of 3.30, 2.85, 2.71, 3.81, 2.11, and 1.94 Å; five of the six were below the 3.5 Å cutoff (Supplementary Figure S7). Their LID–CORE distances ranged from 2.06 to 2.44 nm, close to the closed-reference value of 2.10 nm and substantially smaller than the 3.08 nm distance of the open crystal. Their RMSDs to 4AKE ranged from 5.1 to 7.4 Å.

Following 50 ns of unbiased explicit-solvent MD for each seed, the concatenated trajectory reached a minimum RMSD of 3.31 Å to 1AKE, while closed-like frames accounted for only 0.03% of the total trajectory. Intermediate conformations accounted for 95.5% of frames, with 4.5% classified as open-like. Thus, the closed-like conformations generated by BioEmu were not maintained as a stable closed ensemble during subsequent explicit-solvent MD, nor did they fully relax to the open crystal.

Interpolation MD provided a physics-based comparison. Because the seeds were placed along the Cα interpolation between 4AKE and 1AKE, some initial frames were already within the closed-like region, resulting in a nominal hitting time of 0 ns and 8.3% CC occupancy based on the domain-distance midpoints. After relaxation, the minimum RMSD was 3.06 Å, whereas only 0.37% of frames remained closed-like and 60.1% were open-like. Thus, both interpolation and BioEmu demonstrate that generating or placing a structure near the closed reference does not by itself establish stable closed-state occupancy under the ff14SB/TIP3P Hamiltonian at 300 K. In contrast, TMD maintained the closed state while the target restraint remained active.

### 2.8. Increased CV Coverage Does Not Necessarily Translate into Greater Sampling Efficiency

The computational cost of the different protocols was relatively similar. Unbiased MD, interpolation MD, and BioEmu-relaxation MD achieved approximately 530–534 ns day^−1^, corresponding to about 13.5 GPU h per 300 ns. GaMD, TMD, and simulated tempering required approximately 14.6, 15.9, and 13.9 GPU h, respectively, whereas the three metadynamics protocols required 16.3–16.7 GPU h.

When coverage was normalized by GPU time, functional metadynamics achieved approximately 77 occupied CV bins per GPU hour, compared with 13–19 bins h^−1^ for most other protocols and approximately 30 bins h^−1^ for blind metadynamics. Thus, metadynamics provided substantially greater CV-plane coverage per unit computational cost. However, this gain was accompanied by extensive exploration of over-open conformations. Therefore, higher coverage efficiency should be interpreted as greater exploration of the predefined CV space rather than as evidence of improved thermodynamic sampling or more effective population of the closed functional state.

## 3. Discussion

A same-system, same-budget comparison allows different sampling protocols to be evaluated according to specific scientific questions rather than ranked in the abstract. Under the present conditions, apo AdK provides a stringent test: the closed reference is sufficiently well defined for evaluation, yet sufficiently distant that unbiased MD, GaMD, simulated tempering, and the wrong-CV control did not stably occupy it within 300 ns. This provides a useful baseline for assessing the behavior of the enhanced sampling methods.

When a closed or otherwise functional experimental structure is available, and the objective is to examine a feasible transition pathway, TMD—or related pulling or string methods [17,18]—can directly drive the system toward the target state. In the present study, TMD was the only method that reached and maintained the closed reference, although this behavior should be interpreted as nonequilibrium kinematics rather than equilibrium sampling. When the closed state is unknown and broader exploration is desired, blind metadynamics using *R*_g_ and RMSD to the starting structure provides a CV-light approach, whereas functional domain CVs produce the broadest coverage when the relevant domains are known. In both cases, the trajectories should be examined for over-opening, and the deposited bias should not be interpreted as a quantitative free-energy difference without adequate reweighting and convergence [12]. For sampling a 300 K equilibrium ensemble, unbiased MD remains the physical reference; within the present 300 ns budget, it sampled LID fluctuations and partial closure but did not reach the closed reference. GaMD did not substantially change this behavior, whereas simulated tempering in the 300–400 K range promoted LID opening rather than stable closure. When only sequence information is available, generative models may provide compact candidate structures [1,19,20,21], which should subsequently be evaluated by explicit-solvent MD rather than treated directly as equilibrium ensembles.

The results also support a hybrid workflow that combines sequence-based generation with explicit-solvent simulation. A diverse conformational ensemble can first be generated from sequence using BioEmu [1,19,20,21], followed by selection based on RMSD or domain distances relative to available structural references and relaxation of selected seeds in explicit solvent. In the present AdK system, compact seeds tended to move away from the closed reference during 50 ns of unbiased MD. If occupancy of a known closed state is required, TMD or a related path method provides a more direct approach than unbiased relaxation. Conversely, quantitative free-energy estimation requires a separate simulation campaign involving adequate sampling, reweighting, and convergence assessment [10,11,12]. Unbiased MD remains useful as a physical reference for evaluating spontaneous LID mobility and the stability of generated conformations.

The observation that the LID is more mobile than the NMP domain in unbiased MD, together with the substantial CO and limited OC populations, is consistent with previous studies indicating that LID closure is a relatively accessible component of the apo AdK transition [24,25,26,28,29,30,31,32]. The NMP-first order observed in TMD should therefore not be interpreted as evidence for the equilibrium mechanism of AdK closure. Because the global RMSD restraint acts on all Cα atoms relative to the closed reference, it can recruit the NMP domain before the LID. Path methods that apply different restraints, such as domain-specific targeting or string-based approaches in domain-distance space, may produce different transition orders; these variants were not examined here.

Several limitations should be considered. The work is purely computational: no experimental validation of the sampled ensembles or of the protocol ranking was performed, and the conclusions should therefore be read as provisional with respect to laboratory observables. The study is restricted to wild-type apo AdK and a single force field [33,34]; ligand-bound states and point mutants that alter the open–closed equilibrium were not investigated. The production length was 300 ns rather than the microsecond-to-millisecond timescales associated with many protein conformational transitions, and the metadynamics FES grids were not reweighted. Simulated tempering was likewise not reweighted to 300 K. BioEmu was represented by six relaxed seeds rather than a statistically large generative ensemble, and its model weights were not used as thermodynamic weights. TMD occupancy is restraint-driven, and GPU timings were obtained on a single RTX 4090 using OpenCL and may differ with other hardware or platforms. Finally, the closed reference was defined from ligand-stripped 1AKE, which provides a useful structural reference but is not identical to an experimentally determined apo closed structure.

## 4. Materials and Methods

### 4.1. System Preparation

The open apo structure of *E. coli* AdK, chain A of PDB 4AKE [22], was stripped of crystallographic waters and used as the unique starting coordinates for all physical MD. The closed reference was chain A of PDB 1AKE [23] after removal of the inhibitor AP5A; the ligand was never present in any simulation box. Occupancy of the closed reference is therefore a reference-closed basin defined relative to ligand-stripped 1AKE, not an apo closed experimental structure. Domain definitions followed standard AdK numbering: NMP, residues 30–59; LID, 122–159; and CORE, 1–29, 60–121, and 160–214. Hydrogens were added, the protein was solvated in TIP3P water [35] with 1.2 nm padding, and NaCl was added to 0.15 M. The resulting system contained 39,915 atoms (3341 protein atoms and 12,168 water molecules). The Amber ff14SB force field as distributed with OpenMM 8.6 (amber14-all.xml) was used throughout [33,34,36,37]. A common minimized and equilibrated box (energy minimization, 0.2 ns NVT, 1.0 ns NPT at 300 K and 1 bar) was shared by all subsequent production runs so that method differences are not confounded by independent solvation.

### 4.2. Common MD Settings

Production used a 4 fs timestep with hydrogen-mass repartitioning (1.5 amu) [38], particle-mesh Ewald electrostatics [39], a 1.0 nm nonbonded cutoff, a Langevin thermostat (friction 1.0 ps^−1^), and a Monte Carlo barostat. Protein-only coordinates were stored every 100 ps. Each method was allocated 300 ns of production (TMD ran 308.3 ns because the RMSD restraint schedule slightly overran the target). Simulations were performed with OpenMM 8.6 [36] on an NVIDIA GeForce RTX 4090 (NVIDIA Corporation, Santa Clara, CA, USA) via the OpenCL platform (CPU PME). Throughput was typically 430–535 ns day^−1^ depending on the bias. Minimization and equilibration time are excluded from the 300 ns budget.

### 4.3. Sampling Protocols

Method A (unbiased MD) continued the equilibrated open state for 300.9 ns with no bias. It is the baseline against which every other protocol is judged.

Method B (functional well-tempered metadynamics) [9,10,40] used two CVs: the Cα center-of-mass distances NMP–CORE and LID–CORE. Gaussian hills of height 1.0 kJ mol^−1^ and width 0.05 nm were deposited every 2 ps with a bias factor of 12, on a grid spanning 0.6–4.5 nm. This protocol assumes that the functional domain distances are known.

Method C (targeted MD) applied a harmonic restraint on the Cα RMSD to 1AKE (force constant 8000 kJ mol^−1^ nm^−2^, target 0.05 nm). The target was annealed over the first two-thirds of the run (approximately 200 ns of pulling) and held for the remaining approximately 100 ns. TMD is nonequilibrium: occupancy of the closed reference is not a Boltzmann weight.

Method D (interpolation MD) is a non-generative control for “seeds near the closed reference”. Six Cartesian interpolations between CORE-aligned 4AKE and 1AKE Cα coordinates were built at *λ* = 0.15, 0.32, 0.49, 0.66, 0.83, and 1.00, energy-minimized in stages, and each relaxed for 50 ns of unbiased MD (300 ns total). This is not an AI ensemble; it tests whether placing the system on the open–closed line is sufficient to remain closed in water.

Method E (simulated tempering) [15,16] used eight temperatures geometrically spaced from 300 to 400 K, with attempted exchanges every 2 ps and no CV. The trajectory is a mixture of temperatures (mean 344 K in production) and was not reweighted to 300 K.

Method F (dihedral GaMD) [13,14] collected 2 ns of dihedral-potential statistics on the unbiased system (*V*_min_ = 10,800, *V*_max_ = 11,285, *V*_avg_ = 11,024, *σ* = 75 kJ mol^−1^), then applied a standard GaMD boost (*k*_0_ ≈ 0.107) for 300 ns. No CV and no closed structure were used.

Method G (blind well-tempered metadynamics) replaced domain distances with two CVs that require only the starting structure: the Cα radius of gyration and the Cα RMSD to the equilibrated open coordinates. Hill parameters matched method B. Neither 1AKE nor a domain map entered the bias.

Method H (wrong-CV metadynamics) is a negative control that biases two intra-CORE distances unrelated to LID/NMP opening. Protocol details and results are in Supplementary Section S5.

Method I (BioEmu + MD) used BioEmu, a sequence-based generative ensemble model. Sixteen sequence-only samples were generated with BioEmu v1.1 [1,19,20,21] from the 4AKE FASTA sequence. Six frames were extracted, mapped onto the all-atom topology, and each was relaxed for 50 ns of unbiased MD in the same explicit-solvent box (300 ns total). Generation used the sequence alone; no 4AKE or 1AKE coordinates were provided to the model as a template.

### 4.4. Analysis

All analyses used Cα coordinates. Domain distances are Cα centers of mass. Frames were labeled open or closed if the global Cα RMSD to 4AKE or 1AKE was below 0.35 nm (3.5 Å); remaining frames are intermediate. The crystal open–closed Cα RMSD is 0.71 nm (7.13 Å). Quadrants on the NMP–CORE/LID–CORE plane were split at the midpoints of the two crystal distances (2.02 nm and 2.59 nm), giving OO, OC (NMP closed, LID open), CO (LID closed, NMP open), and CC. Coverage is the number of occupied 0.05 nm × 0.05 nm bins in that plane. A joint principal component analysis (PCA) was performed on CORE-superposed Cα coordinates of all methods plus the two crystals. Cα RMSF was computed after the same CORE superposition onto 4AKE. Hitting times are the first frame below the 3.5 Å closed-reference cutoff; for six-seed methods, the earliest per-seed hitting time is reported. Metadynamics free-energy grids reconstructed from the deposited bias are shown only as biased maps and are not interpreted as Δ*G* [12]. Analysis scripts and numerical tables are in the public repository (https://github.com/liying0128/adk-conformational-sampling (accessed on 18 September 2026)).

During the preparation of this manuscript, the authors used Cursor 3.24 (an AI-assisted writing and coding environment) for language polishing and document formatting. The authors have reviewed and edited the output and take full responsibility for the content of this publication.

## 5. Conclusions

Under a uniform 300 ns explicit-solvent budget, nine conformational sampling protocols for apo *E. coli* adenylate kinase showed distinct sampling behaviors. TMD was the only method that both reached and remained in the closed reference, and it did so under continuous restraint as a nonequilibrium path method rather than as an equilibrium ensemble of the closed state. Well-tempered metadynamics, particularly with functional or blind CVs, provided broader coverage of the domain-distance space, but also promoted LID over-opening and did not yield a reliable free-energy estimate in this study. Unbiased MD, dihedral GaMD, and wrong-CV metadynamics did not produce stable closed-state occupancy, while simulated tempering in a 300–400 K window increased LID opening without producing stable closure. A sequence-only BioEmu ensemble generated closed-like models that were not maintained during subsequent short explicit-solvent MD. Overall, these results show that the ability to explore a CV space, reach a target conformation, and maintain stable target state occupancy represent distinct aspects of conformational sampling, highlighting the importance of matching the sampling strategy to the specific scientific objective. These conclusions are drawn entirely from computation on a single apo enzyme and a single force field. They describe how the tested protocols behave under the present simulation conditions and should not be read as experimentally validated rankings or as equilibrium thermodynamics of AdK; connecting this protocol-to-question mapping to laboratory observables will require experimental tests that were not performed here.

## Figures and Tables

**Table 1:** Comparison of nine sampling protocols under a uniform ~300 ns production budget. Closed-like frames are defined by a Cα RMSD to ligand-stripped 1AKE below 3.5 Å. Occupied CV bins are 0.05 nm cells on the NMP–CORE × LID–CORE plane. LID max is the largest LID–CORE Cα center-of-mass distance (open crystal = 3.08 nm). TMD is the only protocol that both reaches and remains in the closed reference, and it does so under continuous restraint as a nonequilibrium path method rather than as an equilibrium sample. Domain-plane coverage and closed-state occupancy rank the methods differently. Additional descriptors are listed in Table S1.

Method	Min RMSD 1AKE (Å)	Closed-like Frames (%)	CV Bins	LID Max (nm)	Prior Knowledge	GPU h
C TMD	1.28	54.88	213	3.41	Closed reference	15.9
G Blind MetaD	2.43	0.93	494	4.67	*R*_g_ + RMSD to start	16.7
B WT-MetaD	2.89	0.43	1260	4.54	Domain COM CVs	16.3
D Interpolation MD	3.06	0.37	254	3.56	Open + closed references	13.5
I BioEmu + MD	3.31	0.03	247	3.79	Sequence-only	13.6
H Wrong-CV MetaD	3.47	0.03	249	3.66	Intra-CORE distances	16.3
F Dihedral GaMD	3.48	0.03	198	3.66	None	14.6
A Unbiased MD	3.86	0.00	212	3.56	None	13.5
E Sim. tempering	5.83	0.00	268	4.15	*T* ladder 300–400 K	13.9

Methods D and I are concatenations of six 50 ns seeds.

## Data Availability

OpenMM 8.6 run scripts, analysis code, input structures, numerical summaries (summary.json, method_metrics.csv), and figures are available at https://github.com/liying0128/adk-conformational-sampling (accessed on 18 September 2026).

## Supplementary Materials

The following supporting information can be downloaded at: https://www.mdpi.com/article/10.3390/molecules31203686/s1, Section S1: Main-text line plots keep four methods; the remaining traces are shown here; Section S2: Only TMD both reaches and remains in the closed reference in the nine-method overlays; Figure S1: Cα RMSD to 4AKE and 1AKE for all nine methods; Figure S2: RMSD to 1AKE for the five methods omitted from main-text Figure 3; Figure S3: RMSD to 4AKE for the four main-text methods; Figure S4: Coverage growth for all nine methods; Figure S5: Cα RMSF for all nine methods; Figure S6: Joint PCA for all nine methods; Figure S7: BioEmu models before MD relaxation; Figure S8: Simulated tempering ladder occupancy; Figure S9: Wrong-CV metadynamics negative control; Section S3: Hitting times and mixed-quadrant occupancies separate path methods from CV-free runs; Table S1: Additional descriptors not shown in main-text Table 1; Section S4: BioEmu models are closed-like before MD and relax toward open and intermediate states; Section S5: Wrong-CV metadynamics collapses to the unbiased baseline; Section S6: 1 µs unbiased MD and closed-start simulations; Table S2: Additional 1 µs and reverse-TMD runs; Figure S10: Cα RMSD to 4AKE and 1AKE for 1 µs unbiased MD from the closed and open starts; Figure S11: NMP–CORE and LID–CORE distances for closed-start unbiased MD; Figure S12: Cα RMSD to 4AKE for closed→open TMD; Section S7: Domain-colored three-dimensional snapshots; Figure S13: Crystal references, open 4AKE and ligand-stripped 1AKE; Figure S14: Closed-start unbiased MD snapshots; Figure S15: Open-start unbiased MD snapshots; Figure S16: Closed→open TMD snapshots; Figure S17: Domain-colored snapshots of methods D–I.

## Abbreviations

The following abbreviations are used in this manuscript:

AdK	adenylate kinase
BioEmu	biomolecular emulator (generative ensemble model)
COM	center-of-mass
CORE	core domain of AdK
CV	collective-variable
FES	free-energy surface
GaMD	Gaussian accelerated molecular dynamics
HMR	hydrogen-mass repartitioning
LID	lid domain of AdK
MD	molecular dynamics
NMP	NMP-binding domain of AdK
PCA	principal component analysis
PME	particle-mesh Ewald
RMSD	root mean square deviation
RMSF	root mean square fluctuation
TMD	targeted molecular dynamics
WT-MetaD	well-tempered metadynamics
