# Supplementary material for: Comparative Conformational Sampling of Apo Adenylate Kinase by Enhanced Molecular Dynamics and a Sequence-Based Generative Ensemble Model
Source: Molecules. 2026 Oct 14;31(20):3686. doi: 10.3390/molecules31203686 (PMC13707528; doi:10.3390/molecules31203686)
Supplement: Supplementary file 1 [file molecules-31-03686-s001.zip › molecules-4565509-supplementary.pdf]

Supplementary Materials

Comparative Conformational Sampling of Apo Adenylate Kinase by Enhanced Molecular Dynamics and a Sequence-Based Generative Ensemble Model

Qingling Zhao [1,2], Ting Cui [1,2,*] and Ying Li [1,2,*]

1 Library of Beijing Union University, Beijing 100101, China
2 College of Biochemical Engineering, Beijing Union University, Beijing, China
* Correspondence: tsgcuiting@buu.edu.cn (T.C.); liying0128@buu.edu.cn (Y.L.)

S1. Main-Text Line Plots Keep Four Methods; the Remaining Traces Are Shown Here

Main-text line plots keep only the traces needed for the central argument: unbiased MD (A) as the physical baseline, well-tempered metadynamics on functional domain distances (B) as the coverage extreme, targeted MD (C) as the only protocol that both reaches and remains in the closed reference, and blind metadynamics (G) as a CV-light alternative. Interpolation MD (D), simulated tempering (E), dihedral GaMD (F), wrong-CV metadynamics (H) and BioEmu+MD (I) are reported numerically in main-text Table 1 and plotted in Figures S1–S4. Main-text Figure 3 shows RMSD to 1AKE only; RMSD to 4AKE for the same four methods is Figure S3. Protocol, metrics and interpretation of wrong-CV metadynamics (H) follow in Section S5. Additional 1 μs and closed-start simulations, including three-dimensional snapshots, are in Sections S6 and S7.

S2. Only TMD Both Reaches and Remains in the Closed Reference in the Nine-Method Overlays

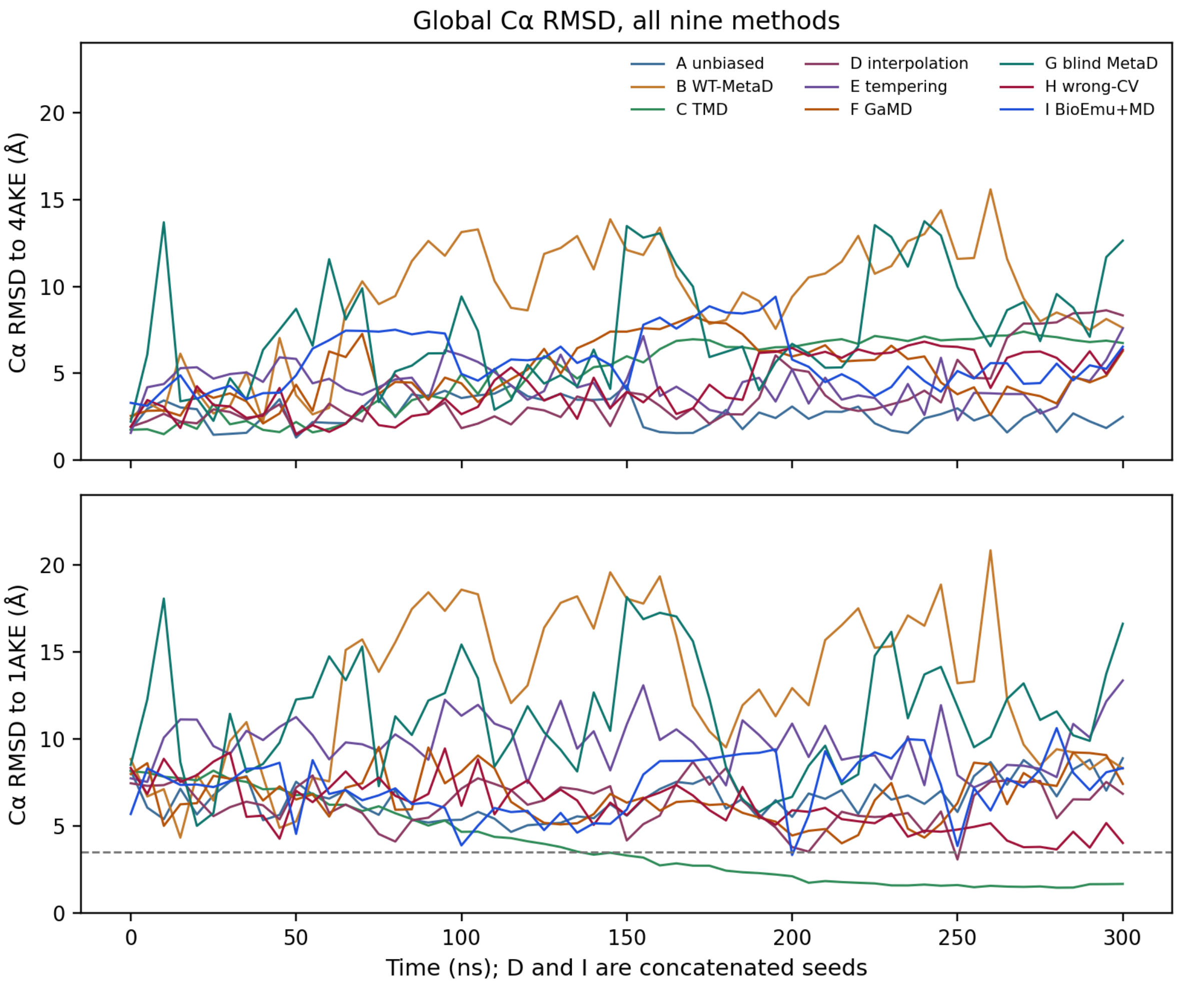

Figure S1. Global Cα RMSD to 4AKE (top) and ligand-stripped 1AKE (bottom) for all nine methods. Frames were saved every 100 ps and are plotted every 5 ns. Methods D and I concatenate six 50 ns seeds. The dashed line in the lower panel is the 3.5 Å closed-reference cutoff used in the main text. Unbiased MD remains near 4AKE; only TMD both approaches and remains near 1AKE.

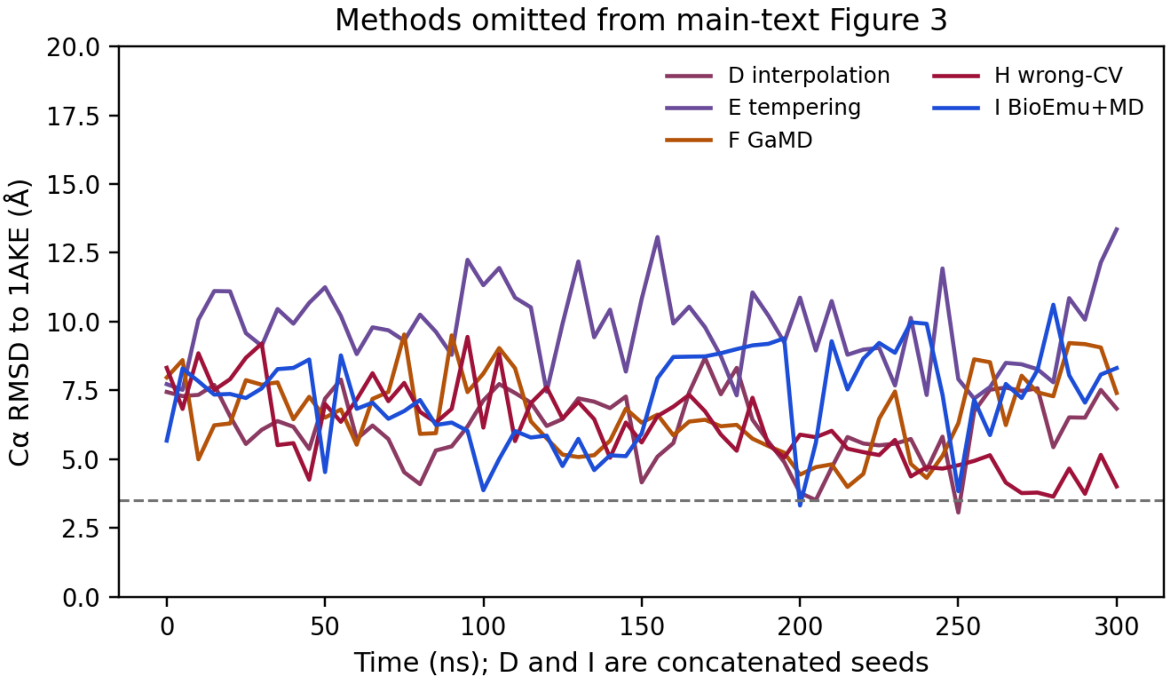

Figure S2. Cα RMSD to the closed reference for the five methods omitted from main-text Figure 3: interpolation MD (D), simulated tempering (E), dihedral GaMD (F), wrong-CV metadynamics (H), and BioEmu+MD (I). None of these methods remains below the 3.5 Å cutoff; simulated tempering is farthest from 1AKE. Methods D and I show brief approaches near seed starts, then relax toward open or intermediate states.

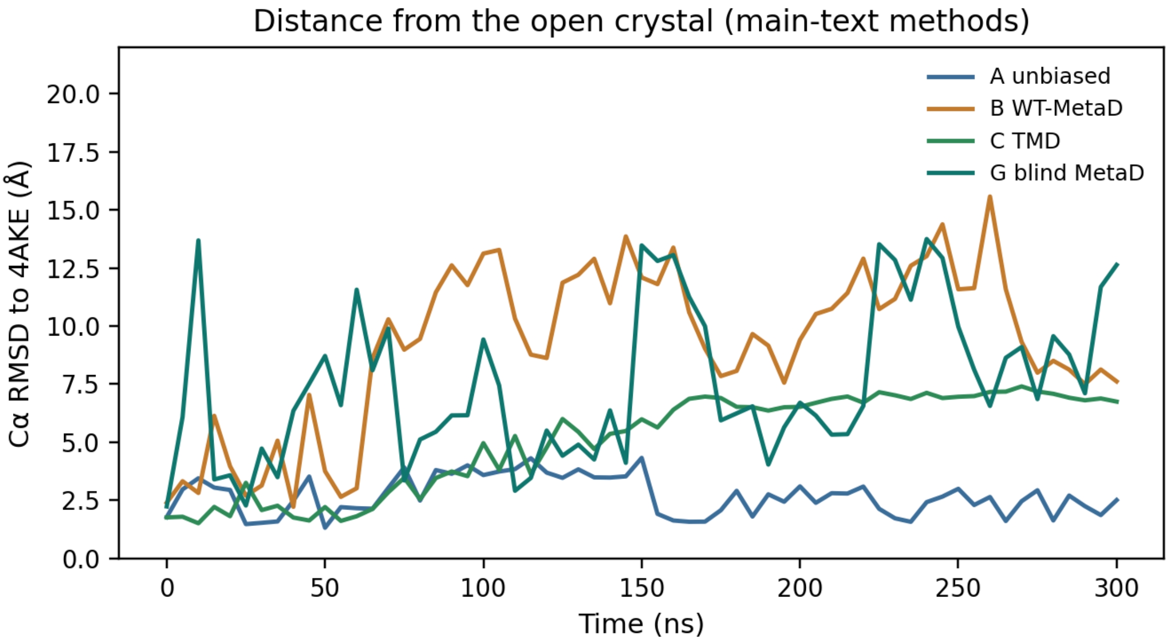

Figure S3. Cα RMSD to open 4AKE for the four methods shown in main-text Figure 3. Unbiased MD remains near the open crystal; TMD leaves 4AKE as it is driven closed; both metadynamics runs drift to large RMSD because the LID over-opens.

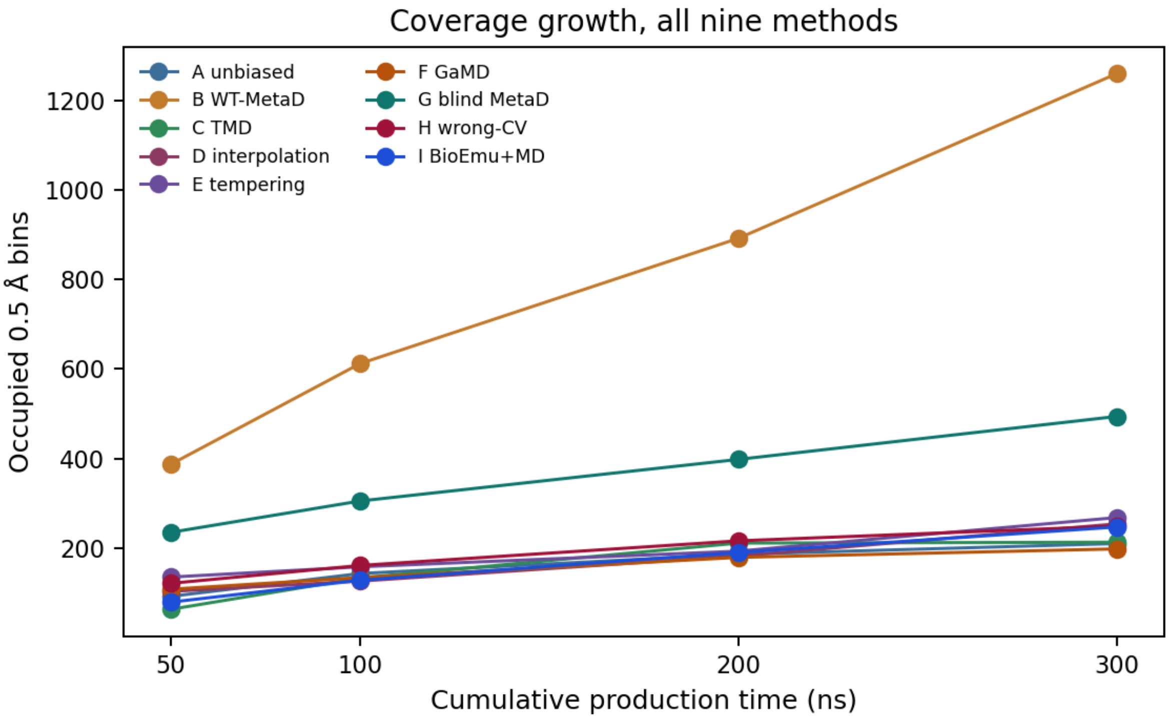

Figure S4. Occupied 0.05 nm bins in the NMP–CORE × LID–CORE plane versus cumulative production time for all nine methods (companion to main-text Figure 2). Functional metadynamics remains the coverage outlier; the additional bins include over-open LID geometries.

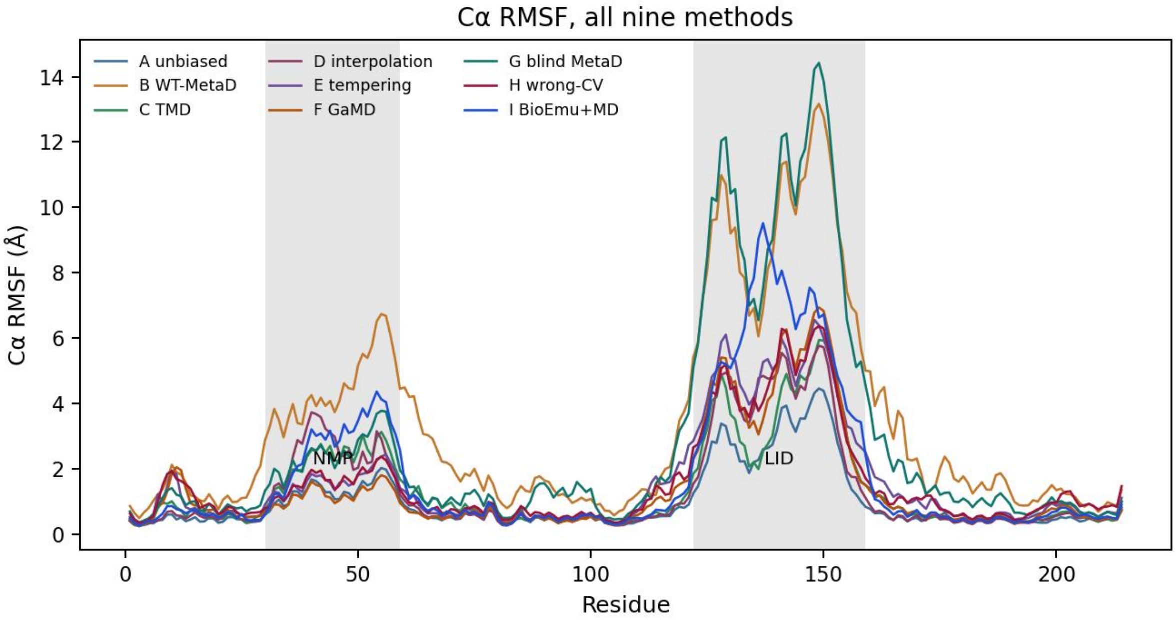

Figure S5. Cα RMSF after CORE superposition onto 4AKE for all nine methods (companion to main-text Figure 7). Blind and functional metadynamics both inflate LID RMSF to approximately 9 Å; simulated tempering and BioEmu+MD increase LID RMSF without reaching the closed reference.

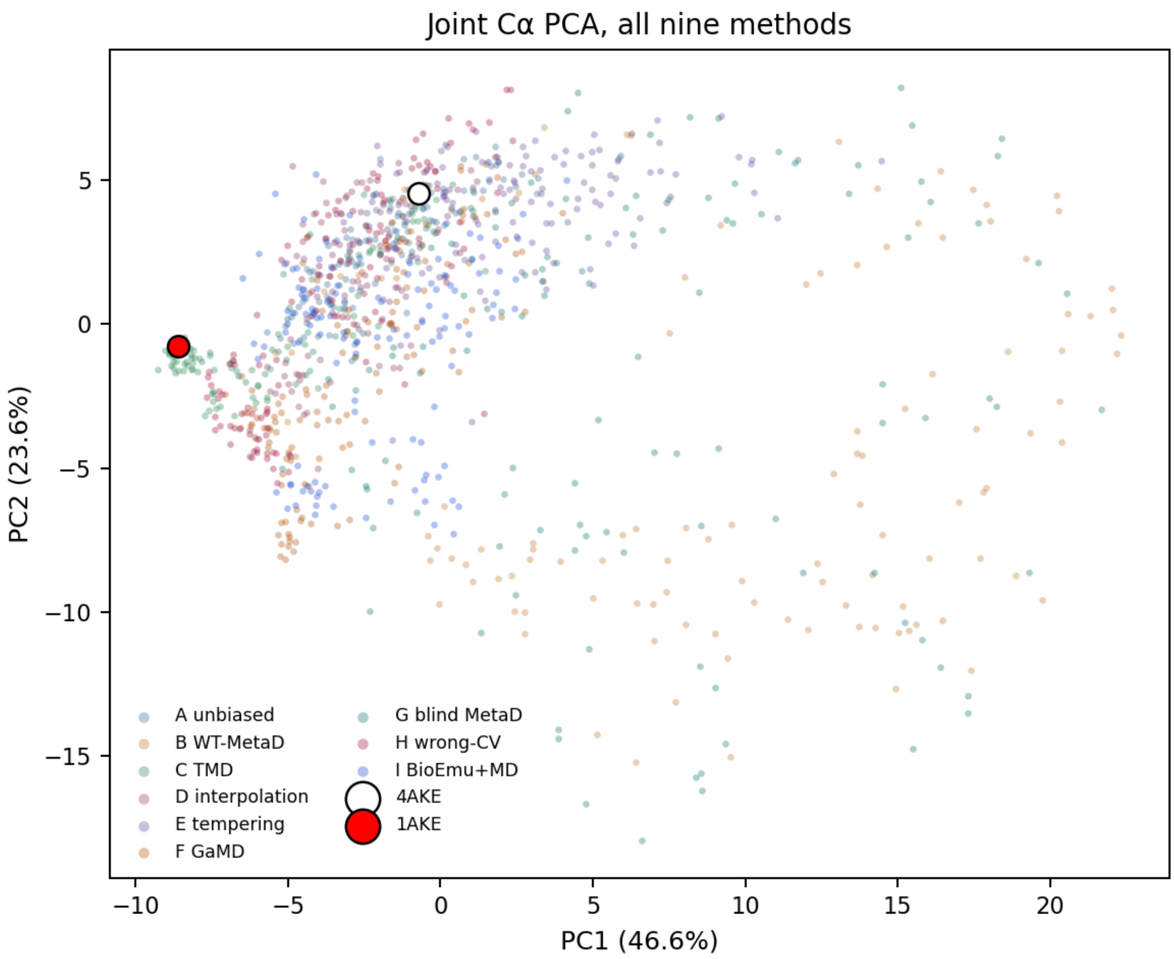

Figure S6. Joint Cα PCA after CORE superposition onto 4AKE for all nine methods (120-frame samples; companion to main-text Figure 6). TMD reaches 1AKE in this plane, whereas metadynamics spreads away from both crystals. White and red markers indicate 4AKE and ligand-stripped 1AKE.

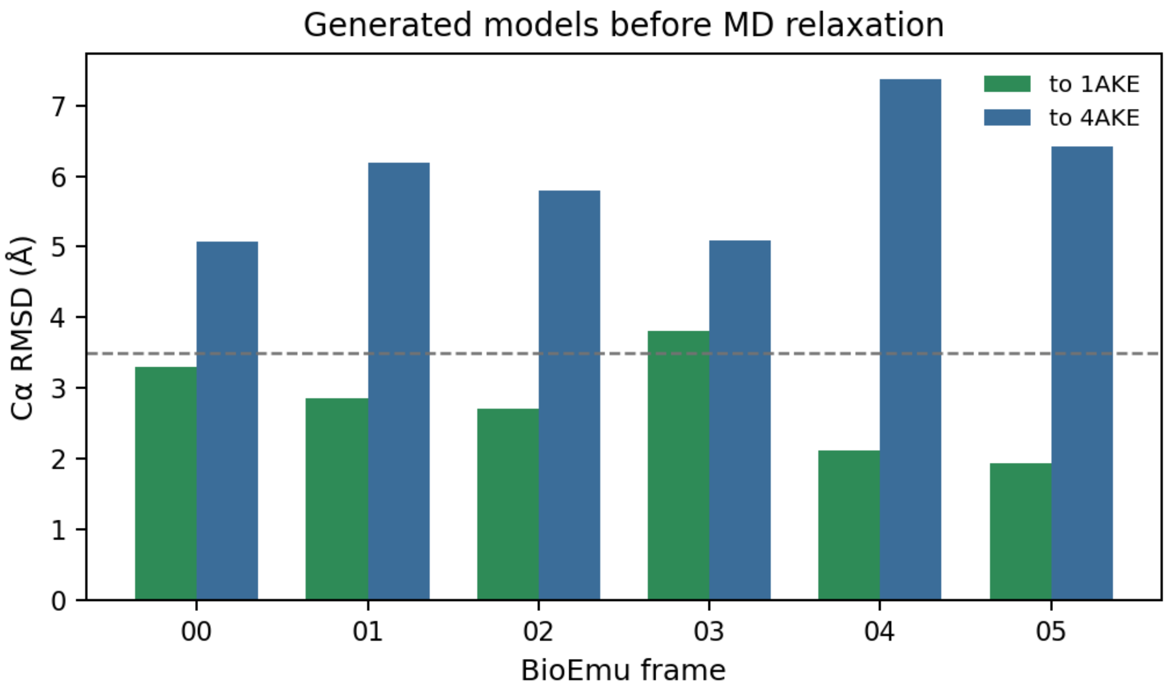

Figure S7. Cα RMSD of the six BioEmu models to 1AKE and 4AKE before explicit-solvent MD (minimum 1.94 Å to 1AKE). Five of the six models are already closed-like. After 6 × 50 ns of unbiased MD, the closest approach of the concatenated trajectory to 1AKE is 3.31 Å (main-text Table 1, method I).

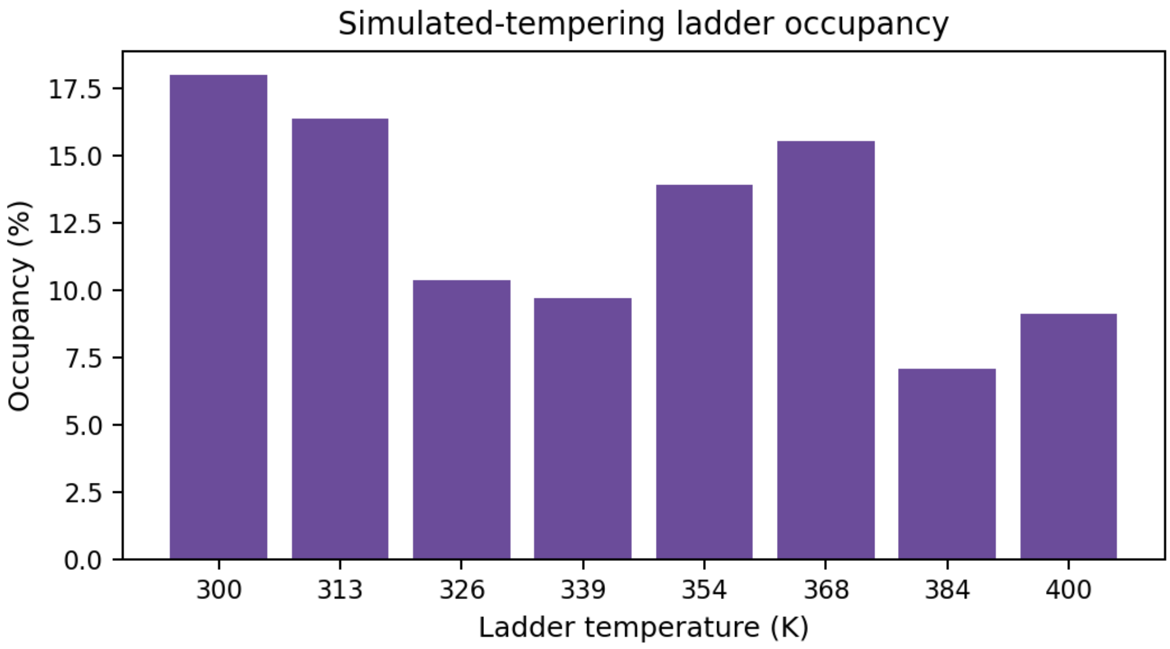

Figure S8. Occupancy of the eight-rung simulated-tempering ladder. The lowest rung (300 K) holds 18.0% of samples and the highest (400 K) 9.1%; intermediate rungs hold 7–16%. The mean production temperature is 344 K. The trajectory is a temperature mixture and was not reweighted to 300 K.

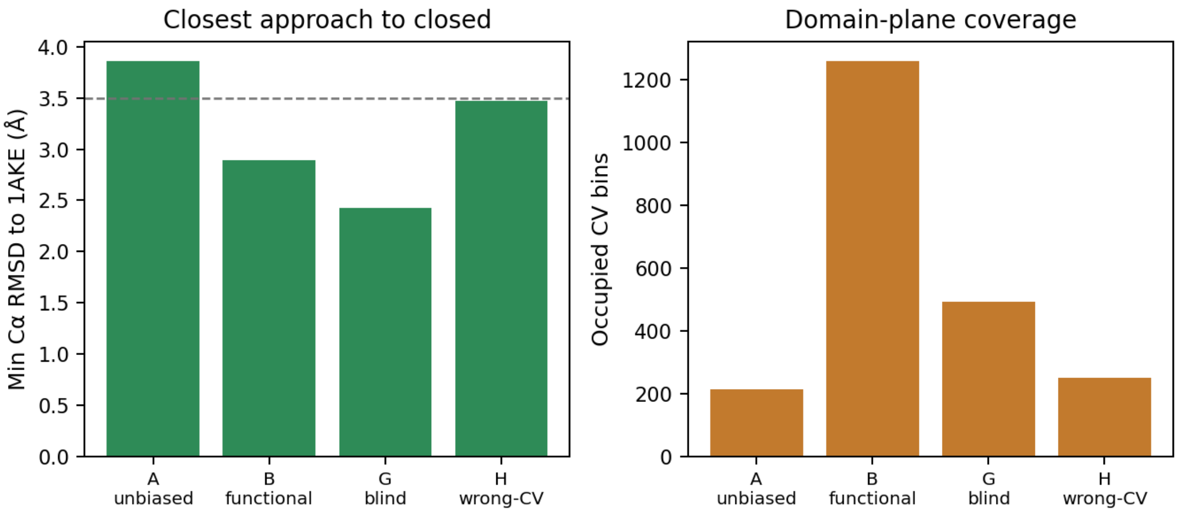

Figure S9. Comparison of wrong-CV metadynamics (method H) with unbiased MD (A), functional metadynamics (B), and blind metadynamics (G). Left: closest Cα RMSD to 1AKE (dashed line, 3.5 Å cutoff). Right: occupied bins on the NMP–CORE / LID–CORE plane. Method H matches unbiased MD on closed RMSD and domain-plane coverage, indicating that a non-functional CV does not promote closing.

S3. Hitting Times and Mixed-Quadrant Occupancies Separate Path Methods from CV-Free Runs

Table S1. Additional descriptors not shown in main-text Table 1. Hit closed is the first time the global Cα RMSD to ligand-stripped 1AKE falls below 3.5 Å (earliest seed for methods D and I). Mixed quadrants are OC + CO on the crystal-midpoint split. Throughput (ns day^{-1}) is the median on one RTX 4090 (OpenCL). These metrics confirm that only TMD both reaches and remains in the closed reference, and only under continuous restraint as a nonequilibrium path method.

Method	Hit closed (ns)	Open / inter. / closed (%)	OO / OC / CO / CC (%)	LID RMSF (Å)	T (K)	ns/day
A unbiased	never	81.5 / 18.5 / 0.00	73.8 / 0.1 / 24.7 / 1.4	2.74	301.0	534
B WT-MetaD	20.8	8.5 / 91.0 / 0.43	69.9 / 2.5 / 26.2 / 1.4	9.03	301.1	441
C TMD	128.7	27.7 / 17.4 / 54.88	28.9 / 0.3 / 18.9 / 51.9	3.64	300.8	465
D interpolation	0 (seed start)	60.1 / 39.5 / 0.37	63.1 / 10.3 / 18.3 / 8.3	3.89	301.0	533
E tempering	never	23.8 / 76.2 / 0.00	98.7 / 1.2 / 0.1 / 0.0	4.70	343.7	519
F GaMD	199.7	23.0 / 76.9 / 0.03	56.0 / 2.4 / 40.1 / 1.4	4.41	301.0	494
G blind MetaD	20.7	8.0 / 91.1 / 0.93	72.0 / 2.0 / 24.8 / 1.1	9.45	301.0	431
H wrong-CV	275.3	44.3 / 55.7 / 0.03	53.2 / 2.3 / 40.9 / 3.6	4.37	301.1	442
I BioEmu+MD	0 (gen. start)	4.5 / 95.5 / 0.03	56.5 / 11.5 / 31.3 / 0.7	5.82	300.9	530

TMD domain order (main text): NMP crosses the crystal-distance midpoint at 24.6 ns, LID at 83.6 ns, RMSD-closed at 128.7 ns. Unbiased histogram FES has no closed reference. The well-tempered metadynamics FES reconstructed from the deposited bias of method B is a biased-potential map (main-text Figure 8) and is not a reweighted ΔG.

S4. BioEmu Models Are Closed-Like before MD and Relax toward Open and Intermediate States

The six extracted BioEmu frames, before MD, have NMP–CORE distances 2.082, 1.895, 1.981, 2.034, 1.975 and 2.063 nm (crystal open 2.23 nm, closed 1.82 nm) and LID–CORE distances 2.203, 2.439, 2.310, 2.330, 2.055 and 2.100 nm (crystal open 3.08 nm, closed 2.10 nm). Cα RMSD to 1AKE: 3.30, 2.85, 2.71, 3.81, 2.11 and 1.94 Å. After 50 ns unbiased MD per seed, closed-like frames drop to 0.03% of the concatenated 300 ns trajectory.

S5. Wrong-CV Metadynamics Collapses to the Unbiased Baseline

Method H is a negative control for collective-variable choice. The two CVs are Cα center-of-mass distances within CORE: residues 8–18 versus 90–100, and 170–185 versus 200–214. Neither pair involves LID or NMP, so the bias cannot encode the open–closed motion. Hill parameters matched methods B and G (height 1.0 kJ mol^{-1}, deposition every 2 ps, bias factor 12). 1AKE and the domain map were not used.

After 300 ns, H is operationally equivalent to unbiased MD for the closed-state question (Figure S9, Table S1): minimum Cα RMSD to 1AKE 3.47 Å (A: 3.86 Å), closed-like frames 0.03%, 249 occupied domain-plane bins (A: 212). LID–CORE still reaches 3.66 nm, comparable to A (3.56 nm) and far below the over-opening of B and G (4.54 and 4.67 nm). Mixed-quadrant occupancy is high (CO 40.9%) because the CORE bias is not inert, but CC remains 3.6% and does not coincide with a stable 3.5 Å RMSD to 1AKE. First hitting time is 275.3 ns—a single late brush of the cutoff.

The practical message is that a plausible-looking but irrelevant CV is not "somewhat useful". It costs the metadynamics overhead (~16.3 GPU hours versus ~13.5 for unbiased MD) and does not buy closing. Blind CVs that at least measure compactness and deviation from the start (G) remain the CV-light option in this panel; functional domain CVs (B) remain the coverage extreme. H should not be interpreted as a failed production method so much as a calibration that CV choice, not the metadynamics algorithm alone, is what expanded the map in B and G.

S6. One-Microsecond Unbiased MD and Closed-Start Simulations

Reviewers asked whether 300 ns is too short for unbiased MD and whether the open→closed ranking is biased because the reverse transition was not tested. We therefore extended the original open-start unbiased trajectory (method A) from 300 ns to 1000 ns by continuing from the saved checkpoint, without modifying the original 300 ns files. Independently, ligand-stripped 1AKE chain A was re-solvated (TIP3P, 150 mM NaCl, same force field and integrator as the main text) and run for 1000 ns of unbiased MD from the closed start. A reverse TMD run (300 ns) used the same harmonic Cα-RMSD protocol as method C (k = 8000 kJ mol^{-1} nm^{-2}, target 0.05 nm, two-thirds anneal then hold) but started from the closed NPT checkpoint and targeted CORE-aligned 4AKE. The closed 1AKE box contains no inhibitor. Analysis used the same 3.5 Å Cα-RMSD cuts and NMP/LID–CORE midpoints as the main text.

Unbiased MD does not complete either full transition in 1 μs (Table S2, Figure S10). From the open start, the closest Cα RMSD to 1AKE is 3.76 Å at 326.5 ns, only 0.10 Å closer than the 300 ns minimum (3.86 Å), and closed-like occupancy remains 0%. From the closed start, the first 300 ns is entirely closed-like. At 364 ns the NMP domain crosses the crystal-distance midpoint and the RMSD to 1AKE drifts above 3.5 Å, but the LID–CORE distance stays between 1.84 and 2.17 nm for the full microsecond (crystal closed 2.10 nm, open 3.08 nm; Figure S11). No frame is open-like relative to 4AKE (minimum RMSD 6.87 Å). The last 100 ns is an NMP-open / LID-closed intermediate, not 4AKE.

Closed→open TMD reaches 4AKE (Figure S12): it first drops below 3.5 Å to 4AKE at 122 ns (minimum 1.11 Å; last 100 ns 100% open-like). The original open→closed TMD is method C in main-text Figure 3 (first drop below 3.5 Å to 1AKE at 129 ns; minimum 1.28 Å; last 100 ns 100% closed-like) and is not repeated here. Reverse TMD opens NMP first (22 ns) and LID later (74 ns), matching the NMP-first order of method C in reverse. Thus, on this apo AdK and this budget, unbiased MD does not occupy the opposite crystal from either end point, whereas TMD does both.

Table S2. Additional 1 μs and reverse-TMD runs, scored with the same 3.5 Å Cα RMSD definition as Table 1. Open-like and closed-like values are frame fractions. Hit times are the first crossing of the cutoff. The open 1000 ns trajectory is a continuation of main-text method A.

Run	Length	Min RMSD 4AKE (Å)	Min RMSD 1AKE (Å)	Open-like (%)	Closed-like (%)	Hit open	Hit closed
Closed unbiased	1000 ns	6.87	2.16	0.0	43.6	never	0 ns
Open unbiased	1000 ns	1.06	3.76	84.1	0.0	0 ns	never
TMD close→open	300 ns	1.11	2.19	57.4	23.2	122 ns	0 ns (start)
TMD open→close (main text C)	300 ns	1.13	1.28	27.7	54.9	0 ns (start)	129 ns

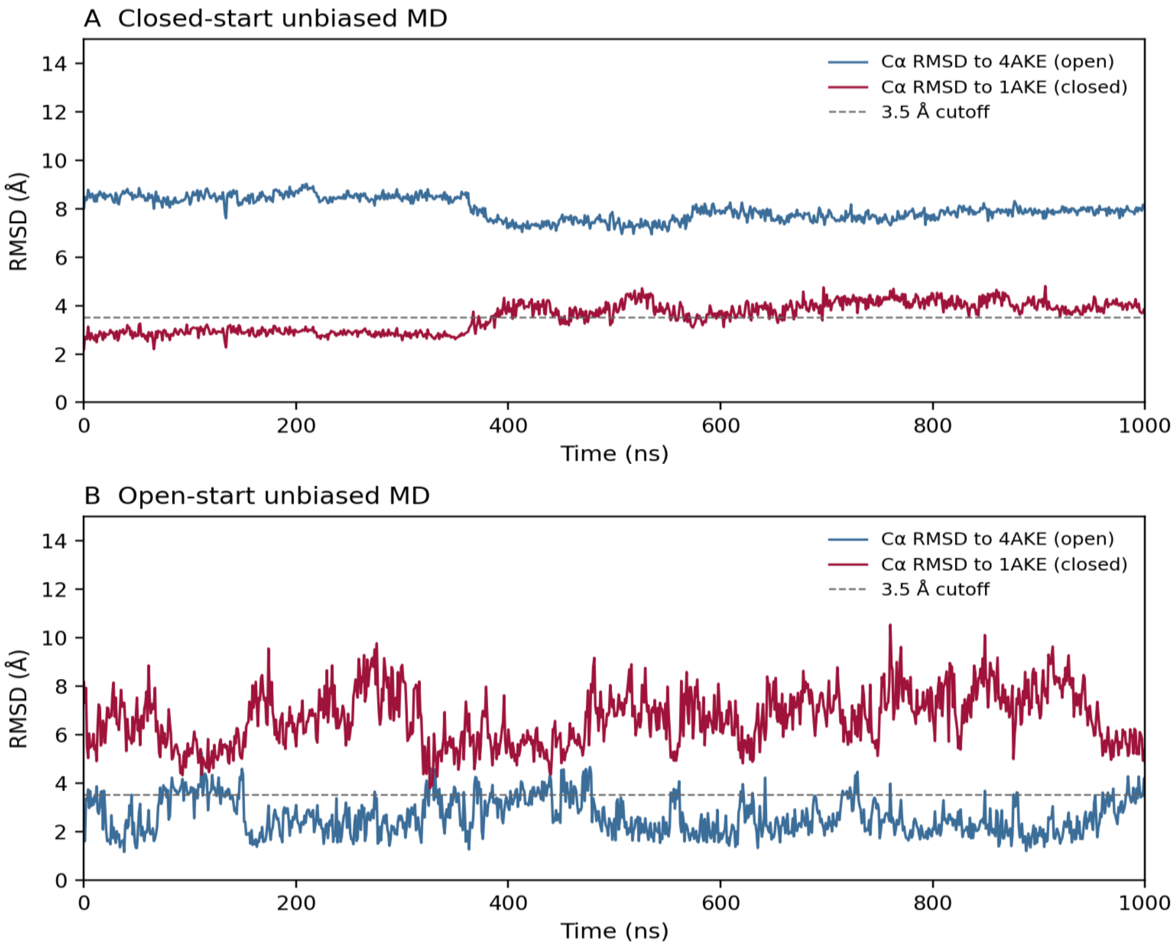

Figure S10. Global Cα RMSD to 4AKE (blue) and 1AKE (red) for 1 μs unbiased MD. (A) Closed start: RMSD to 1AKE remains below 3.5 Å until approximately 365 ns, then drifts above the cutoff without approaching 4AKE. (B) Open start: RMSD to 1AKE never falls below 3.5 Å. The dashed line marks the 3.5 Å cutoff used in the main text. Unbiased MD does not occupy the opposite crystal within 1 μs from either start.

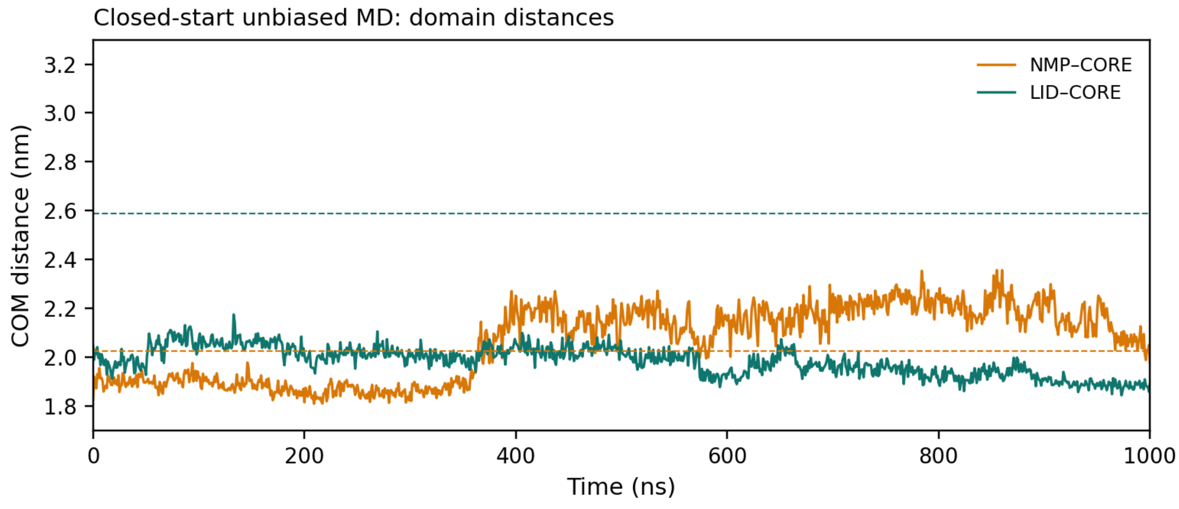

Figure S11. NMP–CORE and LID–CORE Cα center-of-mass distances for closed-start unbiased MD. Dashed lines mark the crystal-distance midpoints used in the main text (2.02 nm and 2.59 nm). The NMP domain loosens after approximately 360 ns, whereas the LID remains shut.

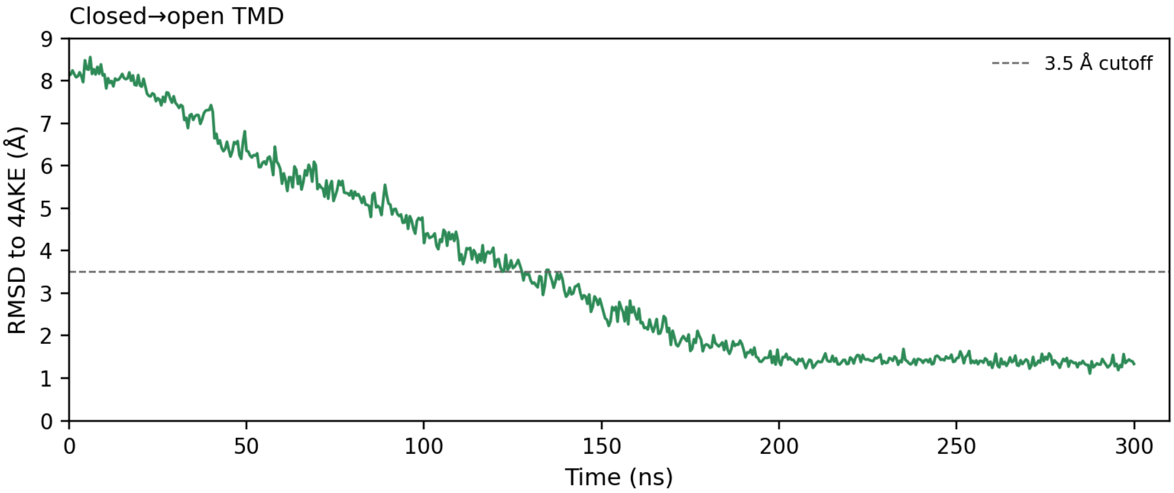

Figure S12. Cα RMSD to 4AKE for closed→open TMD. The dashed line marks the 3.5 Å cutoff. The trajectory reaches 4AKE under continuous restraint. The reverse, open→closed path is method C in main-text Figure 3 and is omitted here to avoid duplicating that panel.

S7. Domain-Colored Three-Dimensional Snapshots

Figures S13–S16 show CORE-aligned cartoons of representative frames, colored by domain (CORE gray, NMP orange, LID teal). The camera is fixed after superposition of CORE Cα atoms onto 4AKE, so LID and NMP displacements are directly comparable across panels. These snapshots are intended to read the time series of Section S6 (Figures S10–S12) on the three-dimensional structure rather than from plots alone. Snapshots from the main 300 ns open-start trajectories of methods A–C are shown in main-text Figure 5. Figure S17 shows CORE-aligned cartoons of the six 300 ns methods not included in main-text Figure 5.

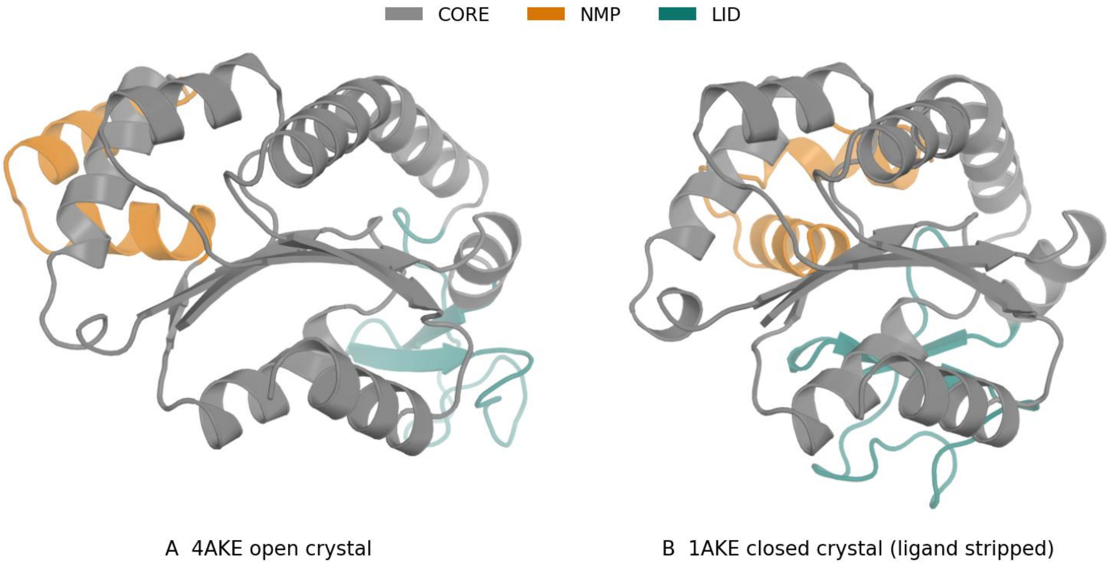

Figure S13. Crystal references used throughout the paper, CORE-aligned. In all panels, CORE is gray, NMP is orange, and LID is teal. (A) Open apo 4AKE. (B) Closed reference: ligand-stripped 1AKE after removal of the inhibitor AP5A. The LID packs against CORE in 1AKE and is displaced in 4AKE.

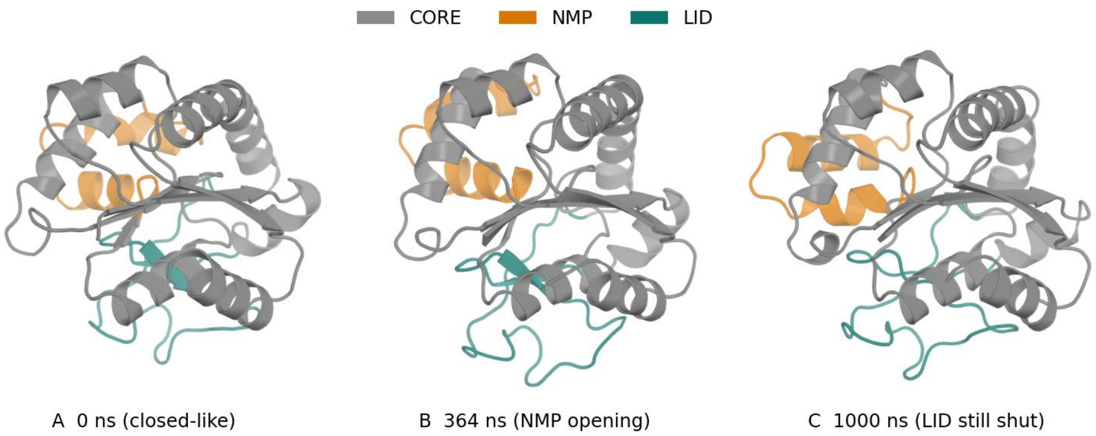

Figure S14. CORE-aligned snapshots of closed-start unbiased MD. In all panels, CORE is gray, NMP is orange, and LID is teal. (A) 0 ns, closed-like. (B) 364 ns, when NMP has crossed the open/closed midpoint. (C) 1000 ns: NMP remains displaced while the LID is still packed against CORE, matching Figure S11. Unbiased MD from the closed start does not open the LID.

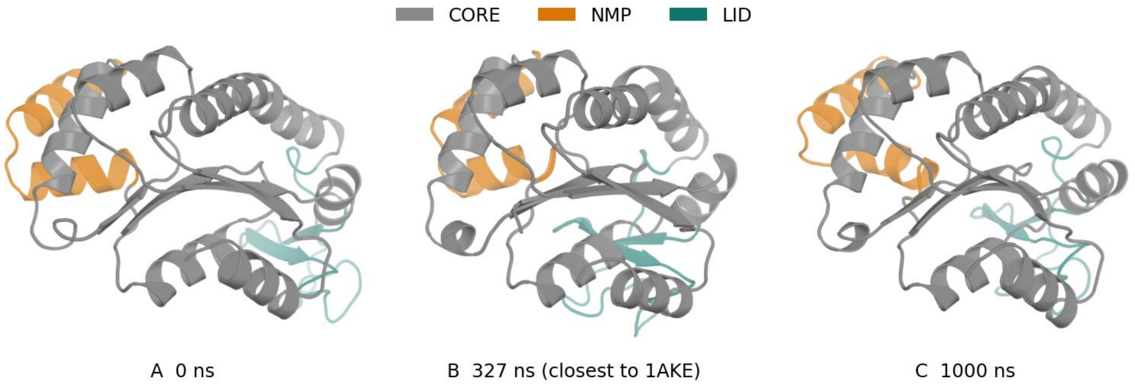

Figure S15. CORE-aligned snapshots of open-start unbiased MD. In all panels, CORE is gray, NMP is orange, and LID is teal. (A) 0 ns. (B) 327 ns, the frame closest to 1AKE (Cα RMSD 3.76 Å, still above the 3.5 Å cutoff). (C) 1000 ns. The LID remains displaced from CORE; the snapshot in (B) is not a closed-reference pose. The trajectory remains open-like at 1 μs.

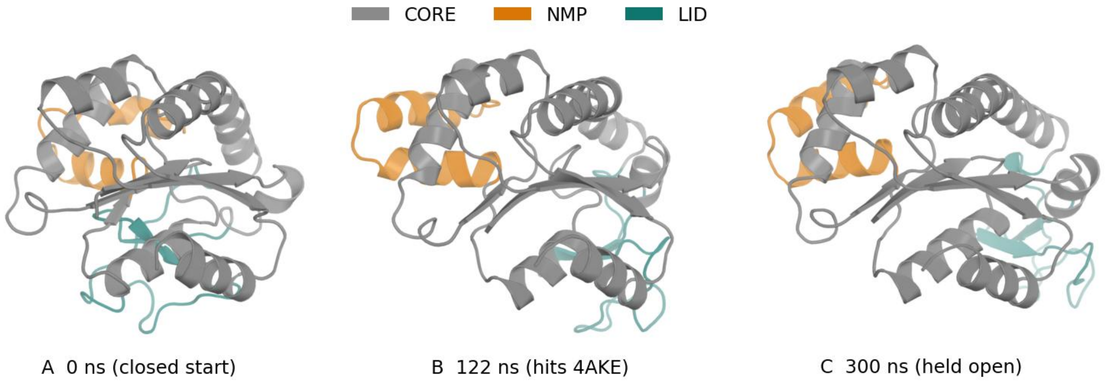

Figure S16. CORE-aligned snapshots of closed→open TMD. In all panels, CORE is gray, NMP is orange, and LID is teal. (A) 0 ns, closed start. (B) 122 ns, first open-like frame (Cα RMSD to 4AKE 3.5 Å). (C) 300 ns, held near 4AKE. Compare with Figure S14C, where unbiased MD from the same closed reference leaves the LID shut.

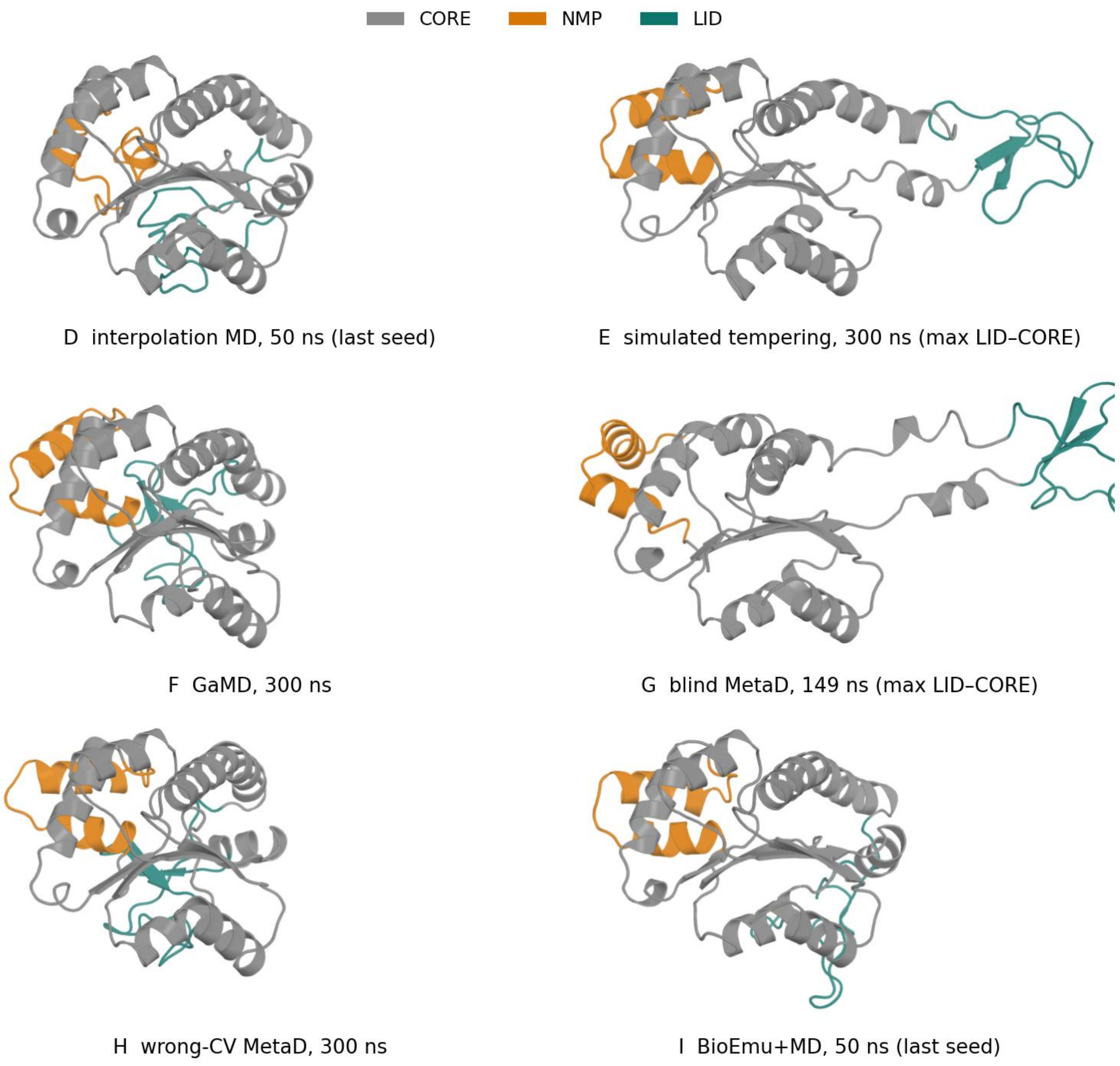

Figure S17. Domain-colored snapshots of methods D–I, CORE-aligned to 4AKE in the same camera as main-text Figure 5. (D) Interpolation MD at the end of the last 50 ns seed (Cartesian $\lambda = 1.00$ start); the compact interpolation does not remain closed. (E) Simulated tempering at maximum LID–CORE distance (4.15 nm at 300 ns); elevated temperature over-opens the LID. (F) Dihedral GaMD at 300 ns remains open-like. (G) Blind metadynamics at maximum LID–CORE distance (4.67 nm at 149 ns), showing LID over-opening as in method B. (H) Wrong-CV metadynamics at 300 ns resembles unbiased MD. (I) BioEmu+MD at the end of the last 50 ns relaxation; generated compact seeds do not remain closed after explicit-solvent MD. CORE, gray; NMP, orange; LID, teal.
